# Halogenated diphenyl ether solvent additives enable ∼20% efficiency organic solar cells and high-performance opaque/semitransparent modules

**DOI:** 10.1093/nsr/nwaf346

**Published:** 2025-08-21

**Authors:** Yibo Zhou, Wenyan Su, Zezhou Liang, Qiang Wu, Hairui Bai, Han Liu, Bohao Song, Xiong Li, Bomin Xie, Chang Liu, Yuchan Zhang, Yu Wang, Jiamin Cao, Xunfan Liao, Guanghao Lu, Yuhang Liu, Ruijie Ma, Huiling Du, Wei Ma, Qunping Fan

**Affiliations:** State Key Laboratory for Mechanical Behavior of Materials, Xi'an Jiaotong University, Xi'an 710049, China; School of Materials Science and Engineering, Xi'an University of Science and Technology, Xi'an 710054, China; School of Materials Science and Engineering, Xi'an University of Science and Technology, Xi'an 710054, China; Key Laboratory of Flexible Optoelectronic Materials and Technology (Ministry of Education), Flexible Display Materials and Technology Co-Innovation Centre of Hubei Province, School of Optoelectronic Materials & Technology, Jianghan University, Wuhan 430056, China; State Key Laboratory for Mechanical Behavior of Materials, Xi'an Jiaotong University, Xi'an 710049, China; State Key Laboratory for Mechanical Behavior of Materials, Xi'an Jiaotong University, Xi'an 710049, China; State Key Laboratory for Mechanical Behavior of Materials, Xi'an Jiaotong University, Xi'an 710049, China; Frontier Institute of Science and Technology, Xi'an Jiaotong University, Xi'an 710054, China; Department of Physics, Beijing Technology and Business University, Beijing 100048, China; State Key Laboratory for Mechanical Behavior of Materials, Xi'an Jiaotong University, Xi'an 710049, China; State Key Laboratory for Mechanical Behavior of Materials, Xi'an Jiaotong University, Xi'an 710049, China; School of Materials Science and Engineering, Xi'an University of Science and Technology, Xi'an 710054, China; State Key Laboratory for Mechanical Behavior of Materials, Xi'an Jiaotong University, Xi'an 710049, China; Key Laboratory of Theoretical Organic Chemistry and Functional Molecule of Ministry of Education, School of Chemistry and Chemical Engineering, Hunan University of Science and Technology, Xiangtan 411201, China; Key Lab of Fluorine and Silicon for Energy Materials and Chemistry of Ministry of Education/National Engineering Research Center for Carbohydrate Synthesis, Jiangxi Normal University, Nanchang 330022, China; Frontier Institute of Science and Technology, Xi'an Jiaotong University, Xi'an 710054, China; State Key Laboratory for Mechanical Behavior of Materials, Xi'an Jiaotong University, Xi'an 710049, China; Department of Electronic and Information Engineering, Research Institute for Smart Energy (RISE), The Hong Kong Polytechnic University, Hong Kong 999077, China; School of Materials Science and Engineering, Xi'an University of Science and Technology, Xi'an 710054, China; State Key Laboratory for Mechanical Behavior of Materials, Xi'an Jiaotong University, Xi'an 710049, China; State Key Laboratory for Mechanical Behavior of Materials, Xi'an Jiaotong University, Xi'an 710049, China

**Keywords:** halogenated diphenyl ether, morphology optimization, organic solar cells and modules, semitransparent devices, power conversion efficiency

## Abstract

Solvent additives are considered as versatile tools to optimize morphology for boosting power conversion efficiency (PCE) of organic solar cells (OSCs). Here, three halogenated diphenyl ether (DPE) solvent additives (fluorinated DPE-F, chlorinated DPE-Cl and brominated DPE-Br) are developed to optimize active-layer (PM6:L8-BO) morphology. With the halogen atomic weight increases, three additives show a gradually increasing boiling point, while DPE-Cl and DPE-Br have similar but a much higher dipole moment compared to DPE-F. The higher boiling point and dipole moment of DPE-Br are expected to enhance the non-covalent interaction between the additive and L8-BO during the active layer film-forming process, offering improved intermolecular packing, charge transport, exciton dissociation and charge collection. As a result, the DPE-Br-treated OSC achieves a higher PCE (18.40%) compared to the DPE-F- and DPE-Cl-treated ones (17.73% and 18.03%). Impressively, using D18 as the donor, the OSCs based on DPE-Br-processed D18:L8-BO:BTP-eC9 obtain a further boosted PCE of ∼20%, while their 11.6 cm^2^ opaque and semitransparent modules also achieve high PCEs of 16.42% and 10.50%, respectively, which are among the top values in OSCs and opaque/semitransparent modules. This work highlights that the halogenation in DPE-derived additives is a promising strategy to optimize morphology for obtaining efficient OSCs and modules.

## Teaser

This work highlights that halogenation in DPE solvent additive is a promising strategy to optimize active-layer morphology and then obtain efficient organic solar cells and modules.

## INTRODUCTION

Organic solar cells (OSCs) are considered to be one of the most promising new energy generators due to their unique advantages of flexibility, semi-transparency and large-scale fabrication [1–11]. Recently, the innovation of non-fullerene small-molecule acceptors (SMAs, including ITIC [12], Y6 [13] and their derivatives [14–21]) and polymerized SMAs (PSMAs, such as ITIC-derived PZ1 [22], Y6-derived PY-IT [23] and their derivatives [24–27]) have greatly improved power conversion efficiencies (PCEs) of OSCs. At present, showing great development and application prospects, the most advanced OSCs with a single-junction active layer have achieved PCEs of more than 19% [28–33]. On the other hand, to achieve the efficient exciton dissociation and charge transport properties for boosting PCEs of OSCs, the active layers combined high-performance wide bandgap polymer donors (such as PM6 [34] and D18 [35]) and near-infrared-absorbing SMAs (such as Y6 [13], BTP-eC9 [36] and L8-BO [37]) are required to form a nanoscale network-interpenetrating morphology with appropriate molecular crystallinity and phase separation [38]. Therefore, precise control of the bulk heterojunction blend morphology of the active

layer has become one of the most important parts in improving photovoltaic performance of OSCs. Up to now, for preparing favourable active-layer morphology, some important strategies have been developed to increase PCEs of OSCs, such as the commonly used thermal and solvent vapor annealing [39], the third component [40], layer-by-layer deposition [41,42], high boiling-point solvent and volatile solid additives [43].

Among the strategies mentioned above, the treatments of high boiling-point solvent additives are considered as the most simple and straightforward process to optimize active-layer morphology of OSCs [44]. By precisely selecting the type and finely tuning the amount of solvent additives, the blend morphology of the active layer can be accurately controlled, and thus construct an ideal nanoscale interpenetrating network structure to fabricate efficient OSCs [45]. Adding solvent additives to optimize active-layer morphology will offer many critical advantages, such as inducing orderly and compactly intermolecular stacking to improve charge transport, controlling the phase separation behavior of active layers to enhance exciton dissociation, and regulating the energy level difference between the excited state of the acceptor component and the charge transfer state in the mixed blend to reduce the voltage loss [46–50]. According to the type of molecular structure, the normally used solvent additives can be mainly divided into the following categories: (i) halogenated alkanes (e.g. 1,8-diiodooctane and diiodomethane) [51,52]; (ii) benzene derivatives (e.g. 1,2-diiodobenzene and diphenyl sulfide) [53,54]; (iii) naphthalene derivatives (e.g. 1-chloronaphthalene, 1-fluoronaphthalene and 1-bromonaphthalene) [55,56]; (iv) thiophene derivatives [e.g. (3,4-dichlorothiophene-2,5-diyl)bis(trimethylsilane) and 2,5-dibromo-3,4-difluorothiophene] [57,58]; and (v) amide derivatives (e.g. *N*-methyl pyrrolidone) [59]. From the frequently used solvent additives mentioned above, it can be confirmed that most of them contain halogen substitutions to increase the boiling point for providing a longer molecular self-assembly time and optimizing the film-forming kinetics process of the active layer, to finally obtain an interpenetrating network structure [60]. So, halogenated solvent additives have been widely proved to synergistically optimize the charge transport and exciton dissociation of OSCs, thereby achieving boosted short-circuit current density (*J*_SC_) and fill factor (FF) simultaneously.

Diphenyl ether (DPE), as one of the most commonly used high boiling-point solvent additives with a long volatilization time, has a strong interaction with active-layer materials, which can finely improve and control the intermolecular stacking and aggregation during the film-forming process [61,62]. Moreover, the advantages obtained above in the DPE active layers tend to lead to a well-distributed interpenetrating network structure in both the lateral and vertical directions, which will improve both the *J*_SC_ and FF of OSCs, as well as minimize the open-circuit voltage (*V*_OC_) loss of OSCs [63]. However, although the reported OSCs with the additional DPE and halogenated organics as solvent additives separately have achieved excellent photovoltaic performance, introducing halogen atoms into DPE additives to more accurately regulate the active-layer morphology has not been reported yet.

Herein, three halogenated solvent additives named DPE-X (including fluorinated DPE-F, chlorinated DPE-Cl and brominated DPE-Br) were developed by attaching different halogen substituents on the parental DPE to optimize active-layer morphology of OSCs. In particular, the effects of introducing DPE-X additives into one of the most advanced binary OSCs, PM6:L8-BO, on the active-layer morphology, and related photovoltaic performance in OSCs were systematically studied. It was found that, with the halogen atomic weight increases, three DPE-X additives offer gradually increasing boiling points, while DPE-Cl and DPE-Br have a similar but much higher dipole moment compared to DPE-F. The higher boiling point and dipole moment of DPE-Br are expected to increase the film-forming time of the active layer and improve the interaction between the additive and L8-BO components, respectively. As a result, among the PM6:L8-BO active layers, the DPE-Br-processed one has a better blend morphology with a more compact and ordered intermolecular packing compared to the DPE-F- or DPE-Cl-processed ones; thus the related devices obtained improved charge transport, exciton dissociation and charge collection behaviors. Therefore, among three OSCs based on PM6:L8-BO, the DPE-Br-treated one achieved a higher PCE of 18.40% compared to the ones treated by DPE-F (17.73%) and DPE-Cl (18.03%). Impressively, using D18 instead of PM6, the DPE-Br-processed OSCs based on D18:L8-BO and D18:L8-BO:BTP-eC9 obtained further boosted PCEs of 19.01% and 19.81%, which are among the top values in binary and ternary OSCs, respectively. Moreover, the 11.6 cm^2^ opaque and semitransparent OSC modules based on DPE-Br-processed D18:L8-BO:BTP-eC9 also achieved excellent PCEs of 16.42% and 10.50%, respectively, ranking among the top values in large-scale OSCs. Further, the above semitransparent OSC module fulfilled an excellent balance of PCE, average visible transmittance (AVT) and thermal insulation properties, making it a good candidate for building integrated photovoltaics. Notably, it was found that DPE-Br outperforms the widely studied solvent additives (Fig. S1 and Table S1) such as alkane derivatives, naphthalene derivatives, benzene derivatives, thiophene derivatives and DPE derivatives, in achieving efficient OSCs. This work highlights that halogenation in DPE solvent additives is a promising strategy to optimize active-layer morphology and then obtain efficient OSCs and modules.

## RESULTS AND DISCUSSION

**Figure 1.**
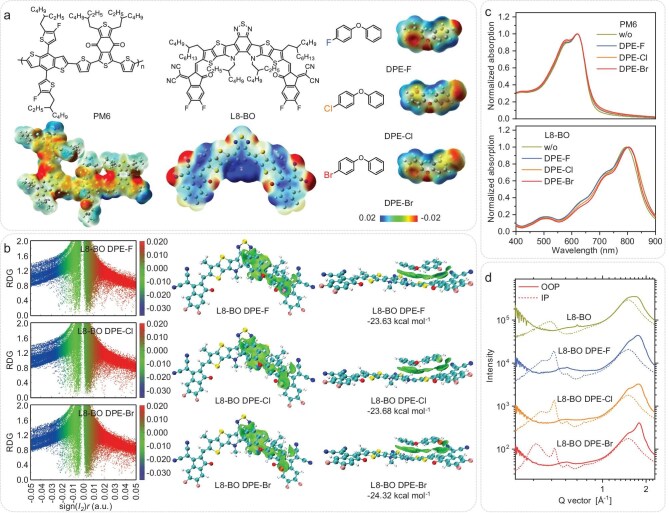
(a) Molecular structures and related ESP patterns of PM6, L8-BO and DPE-X. (b) The functions between RDG and sign(λ_2_), and the corresponding RDG iso-surface maps of L8-BO@DPE-F, L8-BO@DPE-Cl and L8-BO@DPE-Br. (c) Normalized absorption spectra of PM6 and L8-BO films treated without (wo)/with DPE-X. (d) Line-cuts from 2D GIWAXS images of L8-BO films without/with DPE-X processes.

The molecular structures of two active-layer materials (PM6 and L8-BO) and three halogenated DPE-X solvent additives are shown in Fig. 1a. With the increase of atomic weight of halogens, the DPE-X additives show gradually increasing boiling points and gradually decreasing saturated vapor pressures: 149°C/0.37 Pa for DPE-F; 162°C/0.36 Pa for DPE-Cl; and 305°C/0.25 Pa for DPE-Br. Among the three DPE-X additives, DPE-Br has a much higher boiling point and significantly decreased saturated vapor pressure, which prolong the volatilization time of solvents, and thus affect the film-forming dynamic process of active layers and optimize blend morphology (Fig. S2). As shown in Fig. S3a–c, the Fourier transform infrared (FTIR) spectra of the DPE-X-processed PM6:L8-BO blends without/with post-treatment were measured. Before thermal annealing, all the DPE-X-processed PM6:L8-BO blends displayed an obvious FTIR absorption peak at 770 cm^−1^ compared to the additive-free blend, which belongs to the characteristic peak of DPE-X. After thermal annealing at 85°C for 5 min, the characteristic FTIR absorption peaks of DPE-X at 770 cm^−^¹ disappeared in all the blends, confirming that DPE-X additives can be completely removed using thermal annealing. Moreover, the evolution of DPE-Br under thermal annealing was further explored. As displayed in Fig. S3d, under the annealing temperature of 85°C, DPE-Br was removed rapidly and completely within 5 min. The highest occupied molecular orbital (HOMO) and lowest unoccupied molecular orbital (LUMO) energy levels of PM6 [34] and L8-BO [37] were obtained from literature and are summarized in Fig. S4.

The electrostatic potentials (ESPs) of PM6, L8-BO and DPE-X were calculated by density functional theory (DFT). As shown in Fig. 1a, PM6 shows a dominant and continuous negative distribution in conjugated backbone and a dispersed positive distribution in alkyl chains, while L8-BO has almost positive distribution throughout its conjugated backbone and a small amount of scattered negative distribution on thiadiazole and carbonyl/cyano groups [10]. Similarly to PM6, three DPE-X additives exhibit a negative charge distribution, especially the halogen atoms. Therefore, according to the antipolar attraction rule, DPE-X additives are more inclined to form strong intermolecular non-covalent interactions with L8-BO. As depicted in Fig. 1b, the non-covalent interaction between DPE-X and L8-BO can be obtained through a full-space conformational study. In the reduced density gradient (RDG) scatter plots, the red region, representing mutual repulsion, and the blue region, suggesting strong intermolecular attraction, are basically consistent between L8-BO and DPE-X. In contrast, the green region, meaning weak intermolecular attraction, increases slightly in the systems of L8-BO@DPE-F, L8-BO@DPE-Cl and L8-BO@DPE-Br in order, indicating a gradually enhanced non-covalent attractive interaction. Moreover, their binding energy values were calculated. Compared to the L8-BO@DPE-F and L8-BO@DPE-Cl systems with the similar binding energy of −23.63 and −23.68 kcal mol^−1^, the L8-BO@DPE-Br pair offered a higher binding energy of −24.32 kcal mol^−1^. Meanwhile, the Br atom of DPE-X has a bigger atomic radius compared with F and Cl atoms. The increased atomic radius will facilitate spin-orbit coupling (SOC), thereby enhancing the intersystem crossing rate [64]. Therefore, introducing a Br atom into DPE-X is an efficient strategy to enhance the interaction between additive and L8-BO, which helps to achieve more favorable intermolecular π–π stacking and finely control phase separation of the active layer.

As shown in Fig. 1c, compared to the additive-free PM6 films, the DPE-X-treated PM6 films depict almost the same absorption spectra, but slightly increased absorption peaks represent *H*-aggregation, implying weakened intermolecular ordered stacking. In contrast, after introducing DPE-X additives, L8-BO films exhibit a significantly red-shifted absorption peak and absorption onset, indicating an improved π–π interaction, which is consistent with the DFT simulation results. A similar phenomenon has also been found in the PM6:L8-BO blends (Fig. S5). The DPE-Br-treated PM6:L8-BO achieved higher absorption coefficients for both the donor and acceptor components (0.71 × 10^5^/0.77 × 10^5^ cm^−1^) compared to the additive-free DPE-F- and DPE-Cl-treated blends (0.65 × 10^5^/0.70 × 10^5^, 0.68 × 10^5^/0.74 × 10^5^ and 0.69 × 10^5^/0.75 × 10^5^ cm^−1^), which is beneficial for more efficient light harvesting.

To further clarify the effect of halogenation in DPE derivatives on the molecular crystallinity and intermolecular interaction of L8-BO, grazing-incidence wide-angle X-ray scattering (GIWAXS) measurements were conducted (Fig. 1d, Fig. S6 and Table S2) [37]. All L8-BO films show a ‘face-on’ dominated orientation. In the out-of-plane (OOP) direction, additive-free L8-BO film has a (010) diffraction peak located at 1.68 Å^−1^ with a crystal coherence length (CCL) of 12.2 Å, which corresponds to a π–π stacking distance of 3.7 Å. Compared to the control film, all the DPE-X-treated films exhibit a sharper and stronger (010) diffraction peak at 1.78–1.80 Å^−1^ and an additional diffraction peak at 1.49–1.51 Å^−1^, implying an improved intermolecular interaction. For example, after adding DPE-X additives, L8-BO films achieved a smaller π–π stacking distance of 3.5 Å and a higher CCL of 16.1–22.6 Å. Moreover, with the halogen atomic weight increases of DPE-X additives, the related films also obtained gradually improved CCLs and reduced π–π stacking distances. In the in-plane (IP) direction, unlike the additive-free film with a (100) diffraction peak, the DPE-X-treated films depict two independent diffraction peaks. Moreover, among these DPE-X-processed films, the DPE-Br one has higher molecular CCL and smaller intermolecular *d*-spacing values. The results indicate that introducing DPE-X additives can improve intermolecular interaction of L8-BO, especially DPE-Br, which is also highly consistent with their DFT simulation. In addition, to understand the origin of the new diffraction peak located at ∼0.55 Å^−1^ in the GIWAXS of the DPE-X-processed L8-BO neat films, the measurement results between a single crystal of L8-BO and GIWAXS of L8-BO processed without/with DPE-X additives were compared (Fig. S7). Compared to the additive-free L8-BO film, all the DPE-X-processed L8-BO films achieved an obvious diffraction peak located at ∼0.55 Å^−1^ in the IP direction of the GIWAXS measurement, which corresponds to the single crystal measurement of L8-BO with highly ordered stacking. Further, the surface morphology of L8-BO films prepared without and with DPE-X additives was characterized by atomic force microscopy (AFM) measurements. As illustrated in Fig. S8, with adding DPE-X additives, these neat films offer increased root-mean-square (RMS) roughness from 0.48 to 4.02 nm, while the DPE-Br-treated one has the highest roughness, which reflects improved molecular aggregation and is consistent with the absorption and GIWAXS results.

**Figure 2.**
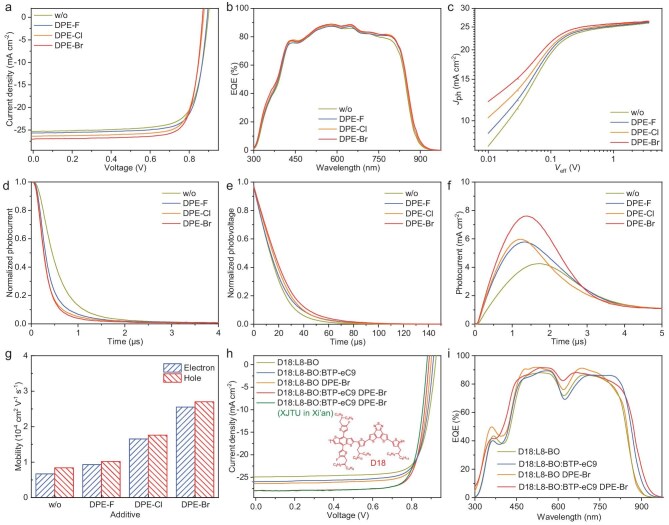
(a) *J–V* curves, (b) EQE plots, (c) *J*_ph__–_*V*_eff_ curves, (d) TPV decay kinetics, (e) TPC decay kinetics and (f) photo-CELIV curves of the OSCs based on PM6:L8-BO without/with DPE-X additives. (g) Summaries of *μ*_e_ and *μ*_h_ values of the PM6:L8-BO-based devices. (h) *J–V* curves and (i) EQE plots of the OSCs based on D18:L8-BO and D18:L8-BO:-BTP-eC9 treated without/with DPE-Br additive. w/o, without.

A device structure of ITO/PEDOT:PSS/PM6:L8-BO/PDIN/Ag was created to study the photovoltaic performance of the OSCs treated without/with DPE-X additives. As shown in Fig. S9 and Table S3, the device performance of OSCs was optimized by adjusting the concentration of DPE-X additives, thermal annealing temperature and spin-coating speed, respectively. The current density–voltage (*J–V*) curves of the optimized OSCs under simulated AM1.5G solar irradiation at 100 mW cm^−2^ are presented in Fig. 2a, and the related photovoltaic parameters are listed in Table 1. The additive-free OSCs achieved a PCE of 17.08% with a *V*_OC_ of 0.898 V, *J*_SC_ of 25.34 mA cm^−2^ and FF of 75.06%. After introducing DPE-X additives, all the OSCs offered higher *J*_SC_ (25.68–26.81 mA cm^−2^) and FF (77.41–79.08%) but a slightly lower *V*_OC_ (0.868–0.892 V), which resulted in boosted PCEs (17.73%–18.40%). The *V*_OC_ values of the OSCs processed from DPE-F, DPE-Cl and DPE-Br decreased in sequence, mainly due to their slightly red-shifted absorption spectra of active layers (Fig. S5), as well as gradually increased energy loss (Fig. S10 and Table S4). Among the DPE-X-processed devices, the DPE-Br-based OSCs gained a lower *V*_OC_ but both higher *J*_SC_ and FF values, achieving a champion PCE, which is mainly due to the optimized active-layer morphology originating from the improved intermolecular interaction between DPE-Br and L8-BO. Furthermore, to confirm the effectiveness of DPE-Br in improving photovoltaic performance, the PM6:L8-BO-based OSCs processed with commonly used liquid additives such as DIO, CN and DPE were prepared as controls. As shown in Fig. S11 and Table S5, the OSCs processed with DIO, CN and DPE delivered PCEs of 17.30%–18.01%, which are higher than the additive-free OSCs (17.08%) but lower than the DPE-Br-treated OSCs (18.4%). Inspired by the strong near-infrared absorption, BTP-eC9 was introduced as the third component into the binary D18:L8-BO blend mentioned above to increase the *J*_SC_ value, thus constructing the efficient ternary OSCs with a further improved PCE. Impressively, using D18 instead of PM6 as donor, the DPE-Br-processed OSCs based on D18:L8-BO and D18:L8-BO:BTP-eC9 obtained further increased PCEs of 19.01% and 19.81% (Fig. 2h), respectively. The above OSCs based on the DPE-Br-processed D18:L8-BO and D18:L8-BO:BTP-eC9 also achieved much higher PCEs compared to their additive-free ones (17.92% and 18.44%), which further confirms the effectiveness of DPE-Br in promoting photovoltaic performance. Additionally, the D18:L8-BO:BTP-eC9-based OSC processed with DPE-Br was fabricated at Xi'an Jiaotong University (XJTU) in Xi'an and also achieved a high PCE of 19.72% (Fig. 2h and Table 1), indicating a good reproduction of BPE-Br additive in preparing efficient devices. For efficient OSCs, it is important to minimize the trade-off between the *J*_SC_ and the *V*_OC_. Therefore, to better compare the device photovoltaic performance of this work and previously reported results, *J*_SC _× *V*_OC_ as the horizontal axis was utilized to summarize the PCEs of the OSCs treated with representative solvent additives. As shown in Fig. S1 and Table S1, DPE-Br outperformed the widely studied solvent additives. To probe the universality of our developed additive strategy in fabricating efficient OSCs, the DPE-X additives were further introduced into the PM1:BTP-eC9 and D18:Y6 systems. As displayed in Fig. S12 and Table S6, all the OSCs based on DPE-X-treated PM1:BTP-eC9 and D18:Y6 offered higher PCEs of 17.54%–17.91% and 18.67%–19.29% compared to the additive-free devices (17.47% and 18.17%).

**Table 1.** Photovoltaic data of the OSCs in this work.

Active layers	Additives	*V*_OC_ (V)	*J*_SC_ (Cal. *J*_SC_)^a^ (mA cm^−2^)	FF (%)	PCE (PCE^avg.^)^b^ (%)
PM6:L8-BO	Without	0.898	25.34 (24.54)	75.06	17.08 (16.96 ± 0.11)
PM6:L8-BO	DPE-F	0.892	25.68 (24.81)	77.41	17.73 (17.52 ± 0.16)
PM6:L8-BO	DPE-Cl	0.874	26.37 (25.08)	78.24	18.03 (17.79 ± 0.17)
PM6:L8-BO	DPE-Br	0.868	26.81 (25.34)	79.08	18.40 (18.22 ± 0.15)
D18:L8-BO	Without	0.922	24.91 (24.03)	78.02	17.92 (17.72 ± 0.19)
D18:L8-BO	DPE-Br	0.902	26.38 (25.23)	79.90	19.01 (18.80 ± 0.18)
D18:L8-BO:BTP-eC9	Without	0.910	25.98 (24.83)	78.02	18.44 (18.19 ± 0.21)
D18:L8-BO:BTP-eC9^c^	DPE-Br	0.887	27.99 (26.74)	79.80	19.81 (19.55 ± 0.20)
D18:L8-BO:BTP-eC9^d^	DPE-Br	0.878	27.94	80.40	19.72

^a^The integrated *J*_SC_ values were calculated from the EQE spectra. ^b^Average PCEs and the deviations calculated from 10 independent devices. ^c^The devices were fabricated at PolyU in Hong Kong. ^d^The device was fabricated at XJTU in Xi'an.

Furthermore, the device stabilities of the OSCs based on PM6:L8-BO processed without/with DPE-X under different conditions were investigated to evaluate their potential practical applications. As shown in Fig. S13, among the abovementioned OSCs, the DPE-Br-treated OSCs obtained similar but slightly superior device stabilities under thermal annealing at 50°C in an N_2_-filled glovebox, 80% ambient humidity in air, and illumination with LED simulated at 1 sun intensity in an N_2_-filled glovebox, respectively, which is mainly due to the optimized morphology caused by the superior non-covalent interactions between DPE-Br and active-layer materials.

To verify the reliability of PCEs, external quantum efficiency (EQE) curves of the above OSCs were measured (Fig. 2b and i). The integrated *J*_SC_ values from EQE curves are 24.54–25.34, 24.03–25.23 and 25.98–26.74 mA cm^−2^ for the OSCs based on PM6:L8-BO, D18: L8-BO and D18: L8-BO:BTP-eC9, respectively, which match their *J–V* measurements very well, with errors of within 5%.

In addition, the photocurrent density–effective voltage (*J*_ph__–_*V*_eff_) curves were plotted to explore the exciton dissociation and charge collection behaviors of OSCs (Fig. 2c) [10,65]. Herein, the probabilities of exciton dissociation (*η*_d_) and charge collection (*η*_c_) of OSCs can be calculated as 97.76% and 86.01% for the additive-free PM6:L8-BO, 97.79% and 87.36% for the DPE-F-treated PM6:L8-BO, 97.95% and 87.67% for the DPE-Cl-treated PM6:L8-BO, and 98.57% and 88.31% for the DPE-Br-treated PM6:L8-BO, respectively. These results indicate that the DPE-Br-processed devices have higher exciton dissociation and charge collection capabilities, which are beneficial for achieving both higher *J*_SC_ and FF values.

To probe the effect of DPE-X additives on the charge recombination of devices, we measured the transient photocurrent (TPC) and transient photovoltage (TPV) of OSCs to explore their charge extraction time and charge carrier lifetime [66]. By fitting the TPC decay curves (Fig. 2d), the decay time of the OSCs with additive-free, DPE-F-, DPE-Cl- and DPE-Br-treated PM6:L8-BO were estimated as 0.48, 0.32, 0.27 and 0.26 μs, respectively, indicating that adding DPE-X additives can significantly improve the charge extraction rate, especially by adding DPE-Cl and DPE-Br. On the other hand, by fitting the TPV decay curves (Fig. 2e), the additive-free, DPE-F-, DPE-Cl- and DPE-Br-processed OSCs achieved gradually increased carrier lifetimes of 18.02, 19.24, 21.29 and 22.92 μs, respectively. The improved charge extraction rate and longer carrier lifetime of the DPE-Br-based OSCs are beneficial for suppressing charge recombination, thus obtaining both higher *J*_SC_ and FF values.

Moreover, the improvement effect of DPE-X additives on the carrier mobilities of OSCs were studied using photo-induced carrier extraction in linearly increasing voltage (photo-CELIV). As shown in Fig. 2f, the DPE-F, DPE-Cl- and DPE-Br-processed devices obtained higher carrier mobilities of 1.78 × 10^−4^, 1.81 × 10^−4^ and 2.05 × 10^−4^ cm^2^ V^−1^ s^−1^, respectively, compared to the additive-free device (0.98 × 10^−4^ cm^2^ V^−1^ s^−1^), which is beneficial for offering superior PCEs. Further, the space-charge-limited-current (SCLC) method was performed to independently probe the charge transport behaviors of devices, including hole and electron mobilities (*μ*_h_ and *μ*_e_). As depicted in Fig. S14, Fig. 2g and Table S7, the additive-free device and devices treated with DPE-F, DPE-Cl and DPE-Br obtained the *μ*_h_ and *μ*_e_ values of 1.51 × 10^−4^/1.09 × 10^−4^, 1.72 × 10^−4^/1.47 × 10^−4^, 2.53 × 10^−4^/2.33 × 10^−4^ and 3.42 × 10^−4^/3.27 × 10^−4^ cm^2^ V^−1^ s^−1^, with *μ*_h_/*μ*_e_ ratios of 1.38, 1.17, 1.09 and 1.05, respectively. Among the above devices, the DPE-Br-treated one had higher and more balanced charge transports, which also partly explains the higher *J*_SC_ and FF values.

**Figure 3.**
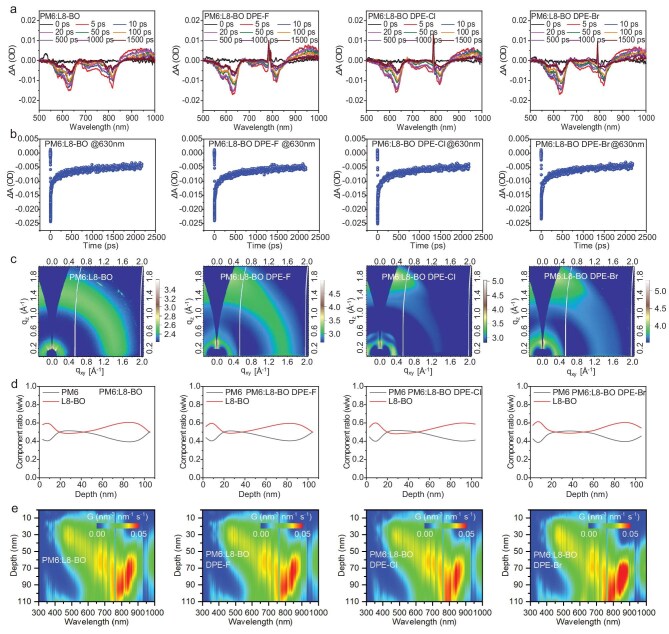
The fs-TAS spectra presented in terms of Δ*T/T*: (a) fs-TAS profiles at different delay times, and (b) electron transfer kinetics and recombination process at 630 nm of PM6:L8-BO blends. (c) 2D GIWAXS profiles of PM6:L8-BO blends. (d) The film-depth-dependent component distributions of PM6 and L8-BO in the blends from FLAS results. (e) Exciton generation contours as numerically simulated from FLAS results.

To deeply understand the detailed photophysical process of the OSCs, time-resolved photo luminescence (TRPL) and femtosecond-resolved transient absorption spectra (fs-TAS) were performed to conduct the general analysis. As shown in Fig. S15, the TRPL spectra of the PM6:L8-BO blends when additive-free or when treated with DPE-F, DPE-Cl and DPE-Br offered the PL lifetimes of 0.169, 0.159, 0.155 and 0.152 ns, respectively. The faster PL lifetime decay of the DPE-Br-processed blend implies superior exciton dissociation in OSCs. The fs-TAS of active layers were measured to study the photogenerated exciton dynamics of OSCs. Figure S16 shows the 2D color plots of fs-TAS of the additive-free, DPE-F-, DPE-Cl- and DPE-Br-processed PM6:L8-BO blends. A pump beam of 790 nm was chosen to solely excite the acceptor due to the significantly different absorption ranges between donor and acceptor components. Negative and positive Δ*T/T* signals correspond to the ground-state bleaching (GSB) and the regions of excited state absorption (ESA) of materials, respectively. As shown in Fig. 3a, a strong GSB peak at ∼820 nm was observed immediately following excitation in all the blends and then the decay of the feature signal with a new GSB peak from the acceptor appears at 630 nm, suggesting the hole transfer from L8-BO to PM6. In Fig. 3b, the electron transfer kinetics and recombination process of active layers are numerically described, via fitting the GSB signal at 630 nm, by the biexponential function with two lifetimes (*τ*_1_ and *τ*_2_). *τ*_1_ is assigned to the ultrafast dissociation time at the D/A interfaces of the excitons formed in the acceptor, and *τ*_2_ is related to the time for excitons moving to the D/A interfaces. Among these blends, the DPE-Br-processed one gained shorter *τ*_1_ and *τ*_2_ values (2.8 and 24.2 ps) compared to the additive-free one (3.9 and 28.6 ps) and those treated with DPE-F (3.7 and 27.1 ps) and DPE-Cl (3.5 and 25.1 ps), suggesting faster exciton dissociation and more efficient exciton diffusion, which is in accordance with its superior FF in OSCs.

Molecular packing, orientation and crystallization behaviors of active layers are the key factors that affect the photovoltaic performance of OSCs. As shown in Fig. 3c, Fig. S17 and Table S8, by carrying out GIWAXS measurements, we explored how DPE-X additives affect the above factors of binary PM6:L8-BO. It can be found that all the active layers show a ‘face-on’ dominated orientation due to their strong (010) diffraction peaks within the range 1.47–1.77 Å^−1^ in the OOP direction and strong (100) diffraction peaks within the range 0.23–0.32 Å^−1^ in the IP direction. Moreover, the DPE-X-treated active layers provide a better ‘face-on’ orientation compared to the additive-free one, while they display gradually improved ‘face-on’ orientation with increasing halogen atomic weight in DPE-X additives, which is beneficial for charge transport along the vertical direction of OSCs. Compared with the additive-free blends, the DPE-X-processed blends exhibit stronger and sharper (010) diffraction peaks in the OOP direction and (100) diffraction peaks in the IP direction, indicating better intermolecular packing and higher crystallinity. In the IP direction, after introducing DPE-X additives to treat active layers, the (100) diffraction peaks shifted from 0.23 to 0.31–0.32 Å^−1^, which corresponds to significantly reduced intermolecular *d*-spacing from 26.9 to 19.4–20.1 Å. In the OOP direction, the (010) diffraction peaks shifted from 1.47 to 1.72–1.77 Å, corresponding to a shortened π–π stacking distance from 4.3 to 3.5–3.6 Å. Notably, these diffraction peaks are more like new diffraction peaks in the optimized morphology by blending components and introducing DPE-X additives, as it is difficult to individually attribute them to PM6 or L8-BO. Moreover, among the DPE-X-treated blends, the DPE-Br-based one stands out due to its higher molecular crystallinity and tighter intermolecular packing, which is also beneficial for charge transports of OSCs.

The AFM images show that all the blend films exhibit fibrous surface morphology (Fig. S18). Among these blend films, the DPE-X-treated blends obtained increased RMS roughness of 0.87–0.97 nm compared to the control blend (0.84 nm), while the DPE-Br blend showed an improved aggregation and higher RMS, which are highly consistent with the GIWAXS results. A similar phenomenon has also been observed in different types of blends. As shown in Fig. S19, the DPE-Br-treated D18:Y6 blend shows a more textured microstructure with an increased RMS roughness of 0.85 nm compared to the additive-free (0.71 nm), DPE-F- (0.75 nm) and DPE-Cl-treated (0.78 nm) blends. The line profiles of full-width half maximum (FWHM) of the peaks are shown in Fig. S20. The average FWHM values were calculated as 6.4–9.3 nm for the DPE-X-treated blends. The DPE-Br blend, with a small FWHM, uniform and size-suitable fibrillary network morphology, achieves both high *J*_SC_ and FF values in OSCs.

Further, the effects of DPE-X additives on the exciton generation of active layers and the vertical phase distributions of PM6 and L8-BO components were studied by measuring the film-depth-dependent light absorption spectroscopy (FLAS) curves (Fig. S21) [67]. As depicted in Fig. 3d, all four PM6:L8-BO blends illustrate a similar component distribution in the vertical direction, while the DPE-Br blend has a more balanced vertical phase distribution of PM6 and L8-BO (especially in a film depth of 20–60 nm). Such an optimized vertical distribution of DPE-Br-treated PM6:L8-BO blend improves exciton dissociation, achieving a higher *J*_SC_ and FF values in OSCs. As shown in Fig. 3e, the exciton generation profiles of active layers can be numerically simulated using the transfer matrix method from the FLAS curves, wherein the generated excitons are mostly concentrated at the bottom of active layers. Among them, the DPE-Br-processed blend has superior exciton generation rates (G) within a film depth of 70–110 nm (Fig. S22). Notably, the G value is the integral result, which represents the cumulative effect in the depth direction. Therefore, the film depth (70–110 nm) with a higher G value may not necessarily be the segment with a more balanced vertical phase distribution (20–60 nm). As a result, the charge of the DPE-Br-treated OSCs can more effectively generate and transport, which supports achievement of a higher *J*_SC_ value. A similar phenomenon has been also observed in the previous reports [68].

To further explore the effect of DPE-X additives on the molecular accumulation of active layers, molecular dynamic (MD) simulation was performed [69], and the related simulation results were shown in Fig. S23. As depicted in Fig. S24a, the centroid-of-mass radial distribution functions (RDFs) of the benzodithiophene (BDT) unit from PM6 relative to L8-BO were extracted from the MD simulations. Compared to the additive-free PM6:L8-BO blend with the first *g*(r) peak at 0.38 nm, the DPE-X-treated blends provide the first *g*(r) peak at 0.28 nm, revealing that the distribution distance of L8-BO around BDT is closer and the probability of L8-BO distribution around BDT increases. Moreover, among the DPE-X-treated blends, the DPE-Br-treated one achieved a higher *g*(r) value of 0.28 nm, indicating that the molecular orientation of L8-BO near the BDT unit is more uniform and the distribution between L8-BO molecules is denser, which helps to enhance intermolecular π–π stacking. Figure S24b depicts the centroid RDFs of L8-BO to L8-BO blend. Among the DPE-X-treated blends, for the first *g*(r) peaks at 0.38 nm, the probability of the distribution around L8-BO slightly increases in the DPE-Br-treated blend. The additive-free blend exhibits a much lower first *g*(r) peak at 0.34 nm compared to the additive-treated blends, and shows concentrated *g*(r) peaks in the centroid distance (r) range of 0.7–1.0 nm, indicating a relatively disordered and loose molecular accumulation.

**Figure 4.**
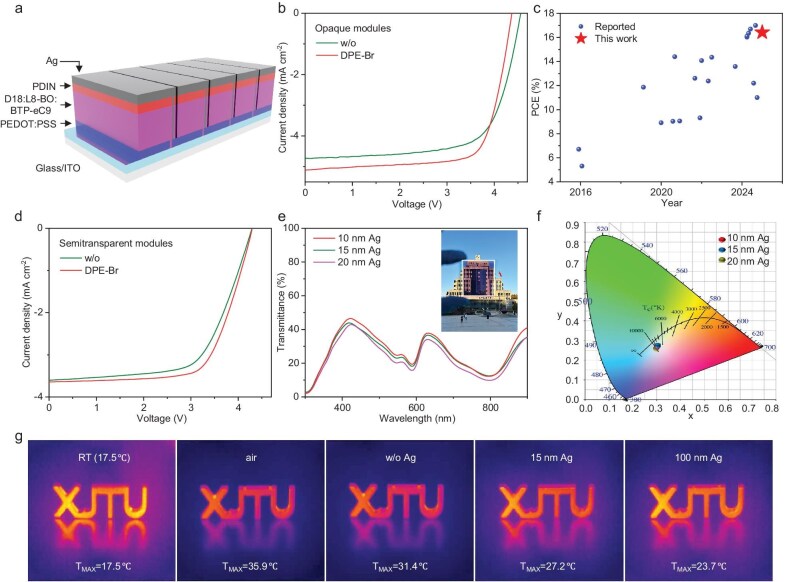
(a) Device structure of the opaque/semitransparent OSC modules based on DPE-Br-treated D18:L8-BO:BTP-eC9. (b) *J–V* curves of the opaque OSC modules. (c) Summary of the PCEs for the opaque OSC modules in this work and reported work. (d) *J–V* curves and (e) transmittance plots of the semitransparent OSC modules, and (f) the corresponding color coordinates on a CIE1931xy chromaticity diagram. (g) Thermal imaging for comparing the thermal insulation effect of semitransparent OSC modules. w/o, without.

To further evaluate the practical application potential of the DPE-Br-treated OSCs, the 11.6 cm^2^ opaque and semitransparent modules based on DPE-Br-treated D18:L8-BO:BTP-eC9 were fabricated, and the corresponding device structure is displayed in Fig. 4a. As shown in Fig. 4b and Table 2, the DPE-Br-treated opaque OSC modules obtained a significantly improved PCE of 16.42% with a slightly decreased *V*_OC_ of 4.37 V but much higher *J*_SC_ of 5.11 mA cm^−2^ and FF of 73.53% compared to the additive-free control (PCE = 14.87%, *V*_OC _= 4.56 V, *J*_SC _= 4.73 mA cm^−2^ and FF = 68.98%). Notably, the PCE of 16.42% is one of the highest values reported in the OSC modules (Fig. 4c and Table S9). A similar phenomenon has been observed in their corresponding semitransparent modules. As shown in Fig. 4d, the DPE-Br-processed semitransparent modules with a 15 nm Ag electrode achieved a boosted PCE of 10.50% compared to the additive-free control with a PCE of 9.79%, while the additive-free and DPE-Br-processed semitransparent OSCs with a smaller device area of 0.038 cm^2^ achieved PCEs of 13.36% and 13.93% with a high light utilization efficiency (LUE) of ∼3.5% (Fig. S25), respectively. As shown in Fig. S26 and Table S10, the D18:Y6-based semitransparent OSCs with a 15 nm Ag electrode and a device area of 0.038 cm^2^ were also fabricated. Compared to the additive-free semitransparent OSCs (12.82%), the DPE-Br-treated ones also achieved a superior PCE (13.62%). Moreover, the DPE-Br-processed semitransparent OSC modules depict excellent transmittance, where the devices with a silver electrode of 10–20 nm thickness offer a high average visible transmittance (AVT) of 23%–27% (Fig. 4e). On the other hand, as illustrated in Fig. 4f of the CIE1931xy chromaticity diagram, the color coordinate of DPE-Br-processed semitransparent modules with a 15 nm Ag electrode is located at (0.305, 0.275), which is close to pure white light. The above high LUE value and good chromaticity coordinate are conducive to their application of DPE-Br-treated semitransparent modules in building integrated photovoltaics [70].

**Table 2.** Photovoltaic data of the opaque/semitransparent OSC modules based on D18:L8-BO:BTP-eC9 treated without/with DPE-Br.

Additive	Ag (nm)	*V*_OC_ (V)	*J*_SC_ (mA cm^−2^)	FF (%)	PCE (%)
Without	15	4.29	3.60	63.32	9.79
Without	100	4.56	4.73	68.98	14.87
DPE-Br	15	4.30	3.64	67.82	10.50
DPE-Br	100	4.37	5.11	73.53	16.42

Finally, the thermal insulation property of DPE-Br-treated multifunctional semitransparent modules was also probed. As shown in Fig. 4g, the DPE-Br-processed modules with a 0, 15 and 100 nm Ag electrode were used as the filter masks on objects, respectively, while an unfiltered object was set as the control. After 5 min of heating under simulated AM1.5G solar irradiation at 100 mW cm^−2^, the temperature of the stainless steel object was measured by thermal imaging. The temperature of the control object changed from 17.5°C to 35.9°C after heating, while the objects using the device filter with 0, 15 and 100 nm Ag showed lower temperatures of 31.4°C, 27.2°C and 23.7 °C, respectively. As a result, our developed DPE-Br-treated semitransparent OSC module with 15 nm Ag realized a great balance between photovoltaic performance and thermal insulation property, making it a good candidate for building an integrated photovoltaic application.

## CONCLUSION

Three halogenated DPE solvent additives (fluorinated DPE-F, chlorinated DPE-Cl and brominated DPE-Br) were developed to optimize PM6:L8-BO morphology. With the molecular weight increases, the three additives displayed gradually improved boiling points, while DPE-Cl and DPE-Br had a similar but much higher dipole moment compared to DPE-F. Therefore, DPE-Br offers enhanced non-covalent interaction with L8-BO to optimize active-layer morphology and improve charge transport and collection of devices. As a result, the DPE-Br processed OSCs achieved a higher PCE compared to the devices treated by DPE-F and DPE-Cl. Impressively, using D18 instead of PM6 as donor, the DPE-Br-processed OSCs provided further boosted PCE approaching 20%, while their 11.6 cm^2^ opaque and semitransparent modules also achieved high PCEs of 16.42% and 10.50%, respectively, which are among the top values in OSCs and opaque/semitransparent modules. Moreover, DPE-Br outperformed the widely studied solvent additives in achieving efficient OSCs. Our work indicates that halogenation in DPE-derived additives is a promising strategy to optimize morphology for obtaining efficient OSCs and modules.

## Supplementary Material

nwaf346_Supplemental_File
