# Supplementary material for: Halogenated diphenyl ether solvent additives enable ∼20% efficiency organic solar cells and high-performance opaque/semitransparent modules
Source: Natl Sci Rev. 2025 Aug 21;12(10):nwaf346. doi: 10.1093/nsr/nwaf346 (PMC12485983; doi:10.1093/nsr/nwaf346)
Supplement: nwaf346_Supplemental_File [file nwaf346_supplemental_file.pdf]

Supporting Information

Halogenated Diphenyl Ether Solvent Additives Enable ~20% Efficiency Organic Solar Cells and High-Performance Opaque/Semitransparent Modules

Yibo Zhou,[a,b] Wenyan Su,[b,] Zezhou Liang,[c] Qiang Wu,[a] Hairui Bai,[a,*] Han Liu,[a] Bohao Song,[d] Xiong Li,[e] Bomin Xie,[a] Chang Liu,[a] Yuchan Zhang,[b] Yu Wang,[a] Jiamin Cao,[f] Xunfan Liao,[g] Guanghao Lu,[d] Yuhang Liu,[a] Ruijie Ma,[h] Huiling Du,[b,*] Wei Ma,[a,*] and Qunping Fan[a,*]*

[a] State Key Laboratory for Mechanical Behavior of Materials, Xi'an Jiaotong University, Xi'an 710049, China. E-mail: baihairui@xjtu.edu.cn; msewma@xjtu.edu.cn; qunping@xjtu.edu.cn
[b] School of Materials Science and Engineering, Xi'an University of Science and Technology, Xi'an 710054, China. E-mail: suwy1027@xust.edu.cn; hldu@xust.edu.cn
[c] Key Laboratory of Flexible Optoelectronic Materials and Technology(Ministry of Education), Flexible Display Materials and Technology Co-Innovation Centre of Hubei Province, School of Optoelectronic Materials & Technology, Jianghan University, Wuhan 430056, China;
[d] Frontier Institute of Science and Technology, Xi'an Jiaotong University, Xi'an 710054, China;
[e] Department of Physics, Beijing Technology and Business University, Beijing 100048, China;
[f] Key Laboratory of Theoretical Organic Chemistry and Functional Molecule of Ministry of Education, School of Chemistry and Chemical Engineering, Hunan University of Science and Technology, Xiangtan 411201, China;
[g] Key Lab of Fluorine and Silicon for Energy Materials and Chemistry of Ministry of Education/National Engineering Research Center for Carbohydrate Synthesis, Jiangxi Normal University, 99 Ziyang Avenue, Nanchang 330022, China;
[h] Department of Electronic and Information Engineering, Research Institute for Smart Energy (RISE), The Hong Kong Polytechnic University, Kowloon 999077, Hong Kong, China
Y. Zhou, W. Su, and Z. Liang contributed equally to this work.

Materials: The polymer donor PM6 (Lot# YN925A) with a M_n of 42.5 kDa and a PDI of 2.2 was purchased from Solarmer Materials Inc. Polymer donor D18 were purchased from Solarmer Materials Inc. Y-series small-molecule acceptors of L8-BO[S1] and BTP-eC9[S2] were synthesized according to the previous reports. Solvent additives of DPE and DPE-X (X = F, Cl, Br) were purchased from Shanghai Aladdin Biochemical Technology Co., Ltd. The boiling points of DPE and DPE-X additives were obtained from network database (www.chemicalbook.com).

Experimental Section

Instruments and Measurements: The UV-vis absorption spectrum and transmittance plots was measured by a Shimadzu UV-1780 Spectrophotometer. Fourier transform infrared (FT-IR) tests were performed on Nicole iN10 of Thermo Scientific Company. Atomic force microscopy (AFM) characterization was scanned by Bruker Atomic Force Microscope using a tapping mode, in which the AFM images have the x-axis and y-axis scales of 2 μm×2 μm and a scale bar of 400 nm. Grazing incidence wide-angle X-ray scattering (GIWAXS) measurements were performed at beamline 7.3.3 at the Advanced Light Source. Samples were prepared on Si substrates using identical blend solutions as those used in devices. The 10 keV X-ray beam was incident at a grazing angle of 0.10-0.16 °, selected to maximize the scattering intensity from the samples. The scattered x-rays were detected using a Dectris Pilatus 2 M photon counting detector. The crystal coherence length (CCL) was defined as CCL=0.9×(2π/FWHM) (Å), where FWHM is the full width at half maximum of the corresponding diffraction peak.

In situ time-resolved UV-vis absorption Measurements: To reveal how the DPE-X additives affect the film-forming time of the active layers, the dynamic processes of film formation were investigated by monitoring the *in-situ* time-resolved UV-vis absorption spectra. The crystallization kinetic behaviors of PM6 and L8-BO during the film formations of PM6:L8-BO blends processed without/with DPE-X can be deduced by monitoring the

temporal change in these absorption intensity, as depicted in **Fig. S2**. The phase-transition processes of the blends can be divided into three stages: I) solvent evaporation, II) crystal growth, and III) stabilize film stages. In stage I, a significant decrease in the absorption intensity of both donor and acceptor can be observed, which is attributed to the removal of excessive solvent. Compared with the PM6:L8-BO blend without additive (0.57 s), the PM6:L8-BO blends with DPE-X showed gradually increased solvent evaporation time (0.62-0.68 s), which facilitates the molecular diffusion and phase separation. In stage II, as the concentration surpasses the solubility, the PM6 and L8-BO enter the crystal growth stage. In the PM6:L8-BO blends, the absorption intensity of L8-BO increases gradually, followed by rapid enhancement of PM6 and L8-BO intensity. The slow increase of absorption intensity of L8-BO corresponds to its pre-aggregation process, while the rapid increases of absorption intensity of donor and acceptor correspond to their crystallization processes. For the PM6:L8-BO blends, in comparison with the additive-free one (0.63 s), the DPE-X treated ones indicated effectively regulated aggregation time of L8-BO (0.47-0.59 s) with an inhibited excessive aggregation, especially DPE-Br treated one, which provides a better foundation for the subsequent crystallization process. In stage III of rapid increase of absorption intensity, the crystallization time of L8-BO in the DPE-X treated blends is basically the same as that of the films without additive, while the crystallization time of PM6 in the DPE-X treated blends increases from 0.09 s to 0.14 s compared to the additive-free blend. This extended duration provided ample time for the formation of ordered molecular packing and phase purity. Subsequently, the acceptor precipitates within the framework formed by PM6, forming an appropriate bi-continuous interpenetrating networks, as demonstrated by AFM characterization, which supports the effective charge generation and extraction in the DPE-Br treated PM6:L8-BO blend.

IR Measurements: The samples for FT-IR testing were prepared under the same conditions as the device fabrication. The concentration was 16.5 mg mL^{-1} in total of PM6:L8-BO. The

PM6:L8-BO (1:1.2, weight ratio) was dissolved thoroughly in chloroform with the DPE-X (0.5%, v/v), respectively, and dissolved for 2 h in a N_2-filled glove box at 80 ℃. The blend solutions were spin-coated on the top of ITO substrates at a speed of 3200 rpm for 40 s, followed by a 85 ℃ for 5 min thermal annealing. Afterwards, the PM6:L8-BO blended materials on ITO were collected and conducted FT-IR testing.

DFT Calculation: The molecular structures were optimized using Gaussian 16 package, under the B3LYP-D3(BJ)/6-31G(d,p) level. To obtained the most possible packing structures between the donor/acceptor materials and the additives, the xtb 6.6.1 and molclus program were employed to search for the lowest energy conformation. First 200 initial conformations through the gentor program were generated by the subroutine gentor program in molclus. All the initial conformations were optimized by xtb 6.6.1 under the gfn1 level, then the five structures with the lowest energies were further structurally optimized using Gaussian16 under the B3LYP-D3(BJ)/6-31G(d,p) level to obtain the structures with the optimal conformations. The reduced density gradient (RDG) analysis by Multiwfn3.8(dev) software.

MD Simulation: The MD simulations were carried out using GROMACS 2022.5 software, using parameters contained its GAFF force field.[3] The cutoffs for van der Waals interactions and for the real-space part of Ewald summation were set at 10 Å. The number of molecules for MD simulations was taken on the basis of the initial experimental feed composition of the investigated systems with the molar ratio mixture of 1:1.2, and convert to number ratio of PM6:L8-BO=100:398, PM6:L8-BO+DPE-X=100:398+40, respectively. The molecules were packed into a box (30×30×30 nm^3) using the Packmol software. The VMD software package was used for the visualization. The velocity rescaling thermostat and the Berendsen barostat under the NPT ensemble were applied to control the temperature and pressure.

(1) PM6:L8-BO: Initially, the system energy was minimized for 1 ns and then gradually heated to the desired temperature 298.15 K for 0.5 ns. And then do periodic annealing 2 times as: heat to 800 k with 0.8 ps/k and maintain with 10 ns, then cooling to 298.15 k with 0.8 ps/k

and maintain with 2 ns. And then, the systems were equilibrated for 5 ns under an NPT ensemble to get the system converge to its experimental condition. Volume averaged over the last 4 ns of isothermal-isobaric ensemble simulations was used to perform canonical ensemble (constant NVT, 298.15 k) simulations of 10 ns, the trajectory data of the last 4 ns was saved at every 5 ps for structural analysis.

(2) PM6:L8-BO DPE-X: The system energy was minimized for 1 ns and then gradually heated to the desired temperature 298.15 K for 0.5 ns. and do periodic annealing 2 times as: heat to 800 k with 0.8 ps/k and maintain with 10 ns, then cooling to 298.15 k with 0.8 ps/k and maintain with 2 ns. And then, the systems were equilibrated for 5 ns under an NPT ensemble to get the system converge to its experimental condition. Volume averaged over the last 4 ns of isothermal-isobaric ensemble simulations was used to perform canonical ensemble (constant NVT, 298.15 k) simulations of 10 ns; then the DPE-X were removed, and 10 ns NVT simulation was further performed, the trajectory data of the last 4 ns was saved at every 5 ps for structural analysis.

Opaque Device Fabrication: The poly(3,4-ethylenedioxythiophene):polystyrene sulfonate (PEDOT:PSS) (Bay PVP. Al 4083, Bayer AG) was filtrated through a 0.45 μm nylon filter, then spin-coated on the cleaned ITO substrates at 5000 rpm for 40 s to form a thin layer (30 nm) and baked at 150 ℃ for 15 min in air. The PM6:L8-BO blends were dissolved in chloroform with the additives (DPE-F, DPE-Cl, or DPE-Br) (0.5%, v/v) under 50 ℃ for 2 hours to mix intensively in a N_2-filled glove box. The concentration was 16.5 mg mL^{-1} in total of PM6:L8-BO. The blend solutions were spin-coated on the top of ITO/PEDOT:PSS substrates at a speed of 3200 rpm for 40 s with an acceleration of 9000 rpm s^{-1}, followed by 85 ℃ for 5 min thermal annealing. The D18:L8-BO (1:1, weight ratio) and D18:L8-BO:BTP-eC9 (1:0.9:0.1, weight ratio) were dissolved thoroughly in chloroform with the DPE-Br (0.5%, v/v), respectively, and dissolved for 2 h in a N_2-filled glove box at 80 ℃. The concentration of D18:L8-BO (1:1, weight ratio) or D18:L8-BO:BTP-eC9 (1:0.9:0.1,

weight ratio) was 9 mg mL^{-1} and the active layer was spin-coated at a speed of 2500 rpm for 40 s with an acceleration of 9000 rpm s^{-1}, followed by a 100 ℃ for 10 min thermal annealing. All the active layers have a thickness of ~110 nm. The PDIN solution (2.0 mg mL^{-1} in methanol with 0.3 vol% acetic acid) was spin-coated on the top of active layers at 5000 rpm for 40 s. Finally, silver electrode (Ag, 100 nm) was deposited under high vacuum (~10^{-5} Pa) in an evaporation chamber. The device area was exactly fixed at 0.038 mm^2.

Semitransparent Device Fabrication: The PEDOT:PSS, active layer, and PDIN were spin-coated on the cleaned ITO substrates as opaque devices, respectively. 15 nm thickness Ag was deposited on the PDIN layer under high vacuum of ~10^{-5} Pa. Then, 35 nm thickness MoO_3 was evaporated onto the surface of Ag.

Large-Area Opaque and Semitransparent Modules Fabrication: The pre-patterned ITO coated glass substrates were purchased from Advanced Election Technology CO., Ltd. The PEDOT:PSS and active layer were fabricated as same as small area devices. And then, the active layers were partially removed by mechanical scribing corresponding to the P2 scribe, for subsequent serial interconnection of the individual solar cells. After that, the PDIN solution (2.0 mg mL^{-1} in methanol with 0.3 vol% acetic acid) was spin-coated onto the top of active layer at 5000 rpm for 40 s. To complete the fabrication of the devices, 100 nm or 15 nm of Ag was thermally evaporated through a mask under a vacuum of ~10^{-5} Pa, during which the each single solar cells were monolithically interconnected.

Device Characterization and EQE measurements: The current-voltage (*J*-*V*) curves were measured on a computer controlled Keithley 2400 Source under AM 1.5G (100 mW cm^{-2}) using a solar simulator (XES-70S1, SAN-EI), which was calibrated by a standard Si solar cell (AK-200, Konica Minolta, Inc.). The light intensity was determined by a 2×2 cm^2 standardized mono silicon cell (Oriel PN 91150V, Newport, USA.) calibrated by the National

Renewable Energy Laboratory (NREL). The EQE values were measured with an EQ-R solar quantum efficiency test system (Enlitech Co., Ltd.).

Stability measurement conditions: All the OSCs used for stability testing were not encapsulated and aged under the following conditions of thermal annealing at 50 ^{o}C (IKA, C-Mag HS7 Digital, heater) in N_2-filled glove-box, 80% ambient humidity (JingYu humidity environment testing equipment, G-D-50L-40) in air, and illumination simulated at 1 sun (Chem^{N}, SLS-LED-80B) in N_2-filled glove-box, respectively.

TPV, TPC, and Photo-CELIV Measurements: Transient photovoltage (TPV), transient photocurrent (TPC), and photo-induce charge extraction linear increasing voltage (Photo-CELIV) were performed using the commercially available Paios system (FLUXiM AG, Switzerland), in which a high-power white LED is implemented as a light source, and the integral power of the LED is 720 W m^{-2}.

Mobility Measurement: The electron or hole mobilities of neat films and blend films were measured by using the method of space-charge limited current (SCLC). The structure of electron-only devices is ITO/ZnO/active layer/PDIN/Ag and the structure of hole-only devices is ITO/PEDOT:PSS/active layer/MoO_3/Ag. The fabrication conditions of the active layer films are same with those for the OSCs. The charge mobilities are generally described by the Mott-Gurney equation (1):

$$J = \frac{9}{8}\varepsilon_r \varepsilon_0 \mu \frac{V^2}{L^3} \quad (1)$$

where J is the current density, ε_0 is the permittivity of free space (8.85×10^{-14} F cm^{-1}), ε_r is the dielectric constant of used materials, μ is the charge mobility, V is the applied voltage and L is the active layer thickness. The ε_r parameter is assumed to be 3, which is a typical value for organic materials. V=V_{app} - V_{bi}, where Vapp is the applied voltage, and V_{bi} is the offset voltage (V_{bi} is 0 V here). The electron mobility can be calculated from the slope of the $J_{0.5}$~V curves.

Time-resolved Photoluminescence (TRPL) Spectra: TRPL was detected using a home-setup microfluorescence system. TRPL decay kinetics were collected using a TCSPC module (PicoHarp 300) and a SPAD detector (IDQ, id100). All the samples were measured in vacuum environments.

Femtosecond-resolved Transient Absorption Spectra (Fs-TAS): The fs-TAS was performed to measure the temporal evolution of the absorption changes in the excited states, through which the carrier dynamics in femtosecond to nanosecond regime could be revealed. The laser beam is supplied by amplified Ti: sapphire laser source (800 nm, Coherent) that provides 100 fs pulses with a repetition rate of 1 kHz. The output was split into two beams, the stronger one of which was frequency doubled to generate a 400 nm pump light, and the other one was focused into a sapphire plate to generate a broadband super-continuum probe light. Using an optical chopper, the repetition rate of the pump pulses was adjusted to 500 Hz, and were focused on the sample with the probe pulse (white light). The TA spectra were obtained by comparing the probe light spectra with and without pump light excitation. The photo-induced absorption change as a function of wavelength was described using optical density (absorbance) changes (ΔOD(λ)). By adjusting the delay time between the pump and probe pulses, a 3D transient spectral image ΔOD(λ,t) was formed.

FLAS Measurements: The FLAS measurements were all conducted by a home-made setup as reported elsewhere under the condition of oxygen plasma pressure below 30 Pa, which generate soft plasma.[5] The FLAS is in-situ measured by a setup which is composed of a halogen light source, a chamber generating soft plasma, and a CCD spectrometer, which are connected by optical fibers.

Average Visible Transmittance (AVT) Calculation: The AVT should be reported as the integration (first moment) of the transmission spectrum and AM 1.5G photon flux weighted against the photopic response of the human eye:[S3]

$$AVT = \frac{\int T(\lambda) \cdot V(\lambda) \cdot AM1.5G(\lambda) d\lambda}{\int T(\lambda) \cdot AM1.5G(\lambda) d\lambda} \tag{2}$$

where $T(\lambda)$ is the transmission spectrum and $V(\lambda)$ is the photopic response. It is estimated by taking the average of the transparency of the devices in the visible region (380-740 nm) based on the photonic response of the human eye.

Thermal Insulation Performance Test: The modules were heated under the standard AM 1.5G (100 mW cm^{-2}) using a solar simulator, and the heat distance is 50 cm. The thermal images were shot by CEM DT-9897H.

Energy Loss Measurements: To probe why the V_{OC} decreased in sequence of the OSCs processed from DPE-F, DPE-Cl, and DPE-Br, their energy loss (E_{loss}) were analyzed. The total E_{loss} of devices was assessed by the equation of E_{loss} = E_g^{PV} - qV_{OC}, where E_g^{PV} is the optical bandgap that can be determined from the derivatives of the highly sensitive fourier transform photocurrent spectroscopy EQE (FTPS-EQE) of the devices. The E_g^{PV} of the devices based on PM6:L8-BO processed with additive-free, DPE-F, DPE-Cl, and DPE-Br are 1.457, 1.453, 1.450, 1.449 eV, respectively. Therefore, the total E_{loss} was calculated to be 0.581 eV for the OSCs treated with DPE-Br, which is higher than those of ones processed with additive-free (0.559 eV), DPE-F (0.561 eV), and DPE-Cl (0.576 eV). Generally, the total E_{loss} can be divided into three parts: the radiative recombination loss above the bandgap (ΔE_1), the radiative recombination loss below the bandgap (ΔE_2), and the non-radiative recombination loss (ΔE_3), respectively. ΔE_1 can be defined as E_g^{PV} - qV_{OC}^{SQ}, where qV_{OC}^{SQ} is the maximum theoretical V_{OC} derived by Shockley-Queisser model. All the OSCs processed without/with DPE-X achieved a similar ΔE_1 of 0.258 eV. ΔE_2 can be calculated by qV_{OC}^{SQ}- qV_{OC}^{rad}. The V_{OC}^{SQ} values were determined to be 1.155, 1.140, 1.112, and 1.107 V for the OSCs processed with additive-free, DPE-F, DPE-Cl, and DPE-Br, which correspond to the ΔE_2 values of 0.044, 0.055, 0.080, and 0.084 eV, respectively. According to the formula of

ΔE_3 = qV_{OC}^{rad}- qV_{OC}, the ΔE_3 values were measured as 0.257, 0.248, 0.238, and 0.239 eV for the OSCs processed with additive-free, DPE-F, DPE-Cl, and DPE-Br, respectively. On the other hands, the experimental ΔE_3 values can be also calculated by the equation of -kTln(EQE$_{EL}$). As a result, among these devices, the DPE-Br treated one achieved the highest total E_{loss}.

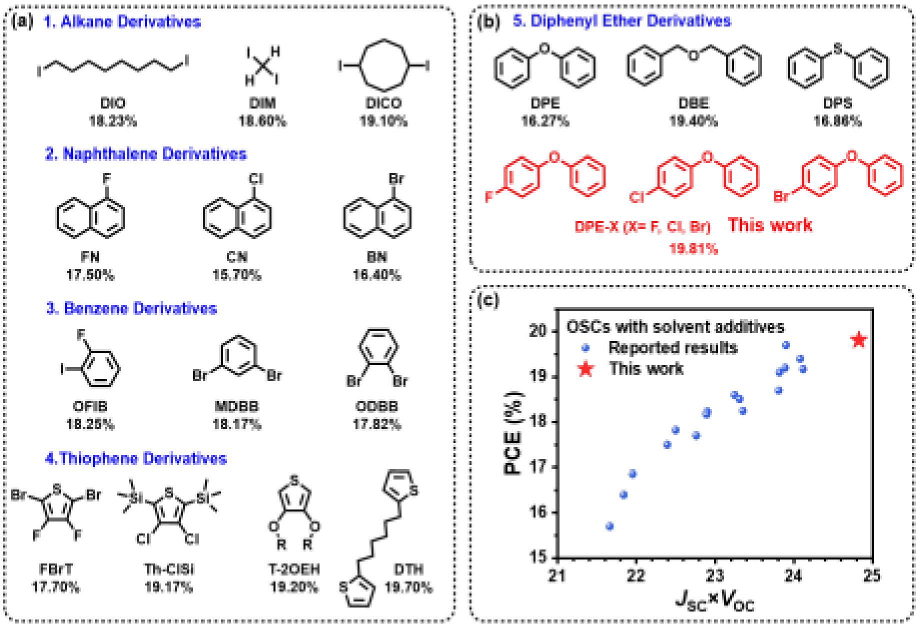

Fig. S1 (a and b) Chemical structures of the representative solvent additives used in the previous reports and this work. (c) Summary of the PCE values versus $J_{SC} \times V_{OC}$ values for the state-of-the-art OSCs processed with solvent additives reported in this work and the literature.

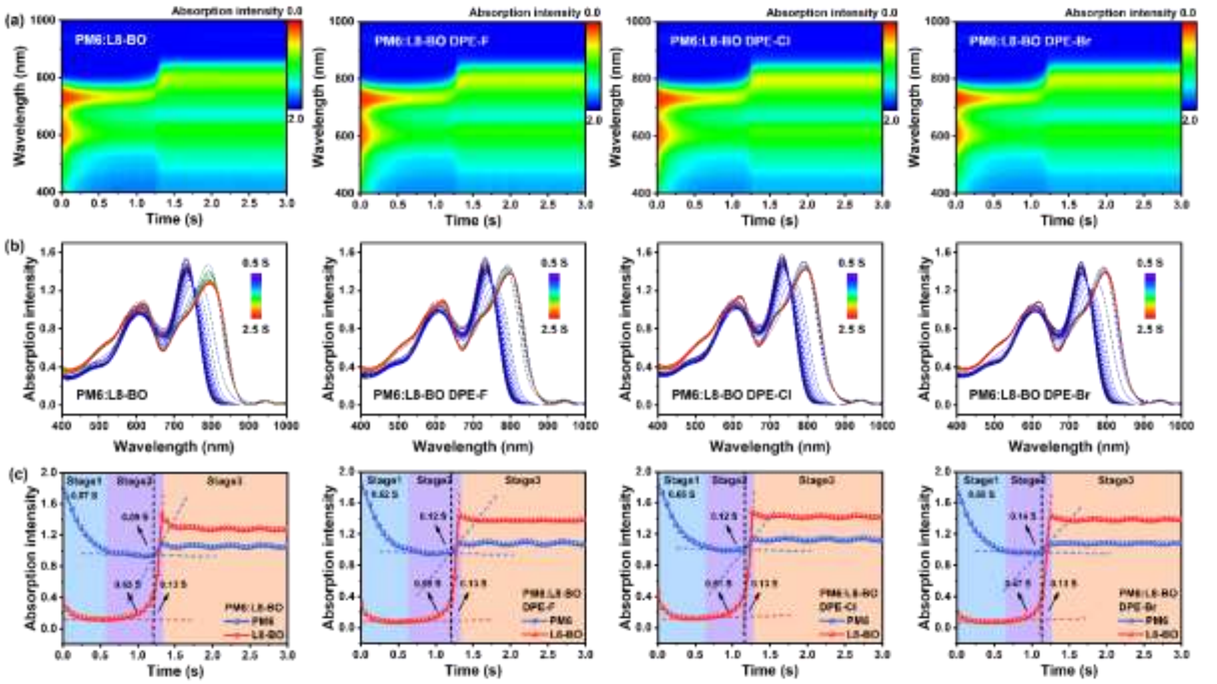

Fig. S2 For the PM6:L8-BO blends processed without/with DPE-X: (a) The time-dependent contour plots of *in-situ* UV-vis absorption spectra, (b) the absorption spectra at different times, and (c) the peak intensity time evolution of PM6 and L8-BO absorption, respectively.

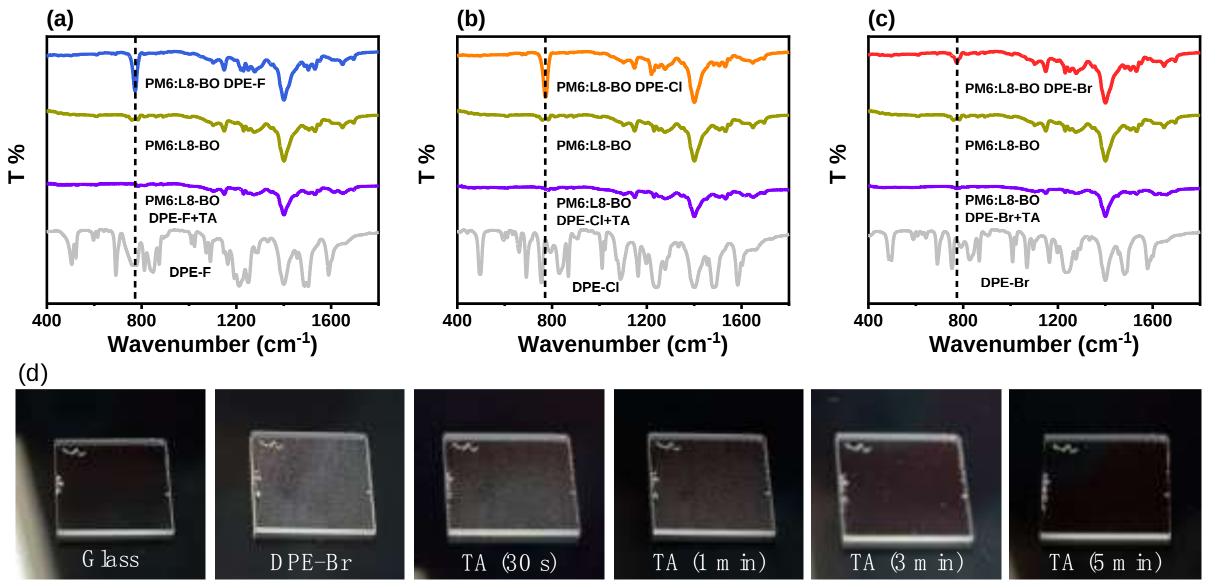

Fig. S3 (a-c) Fourier transform infrared (FTIR) spectra of the PM6:L8-BO blends without/with DPE-X and thermal annealing treatments. (d) Evolution of DPE-Br on the glass under thermal annealing at 85 ℃.

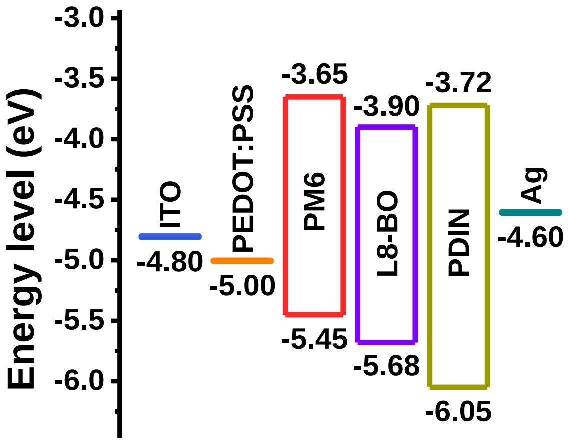

Fig. S4 The diagrams of HOMO and LUMO levels of materials.

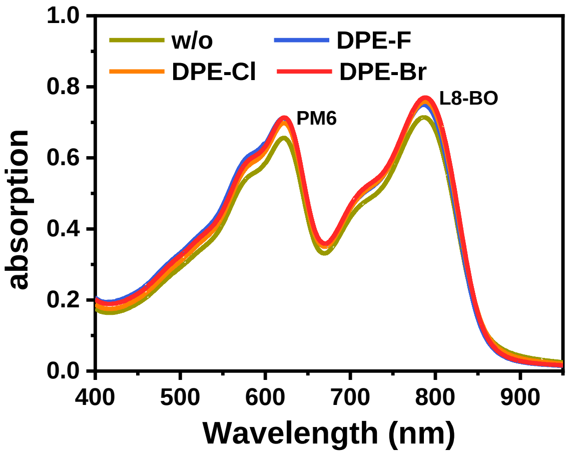

Fig. S5 Absorption spectra of PM6:L8-BO blends processed without/with DPE-X additives.

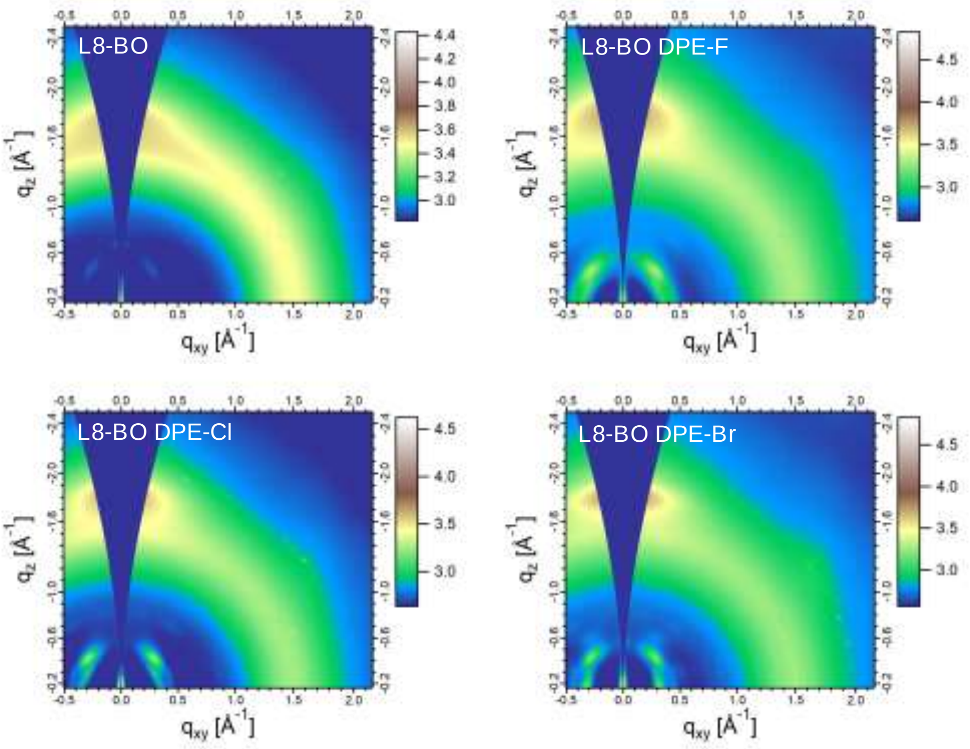

Fig. S6 2D GIWAXS images of L8-BO films treated without/with DPE-X additives.

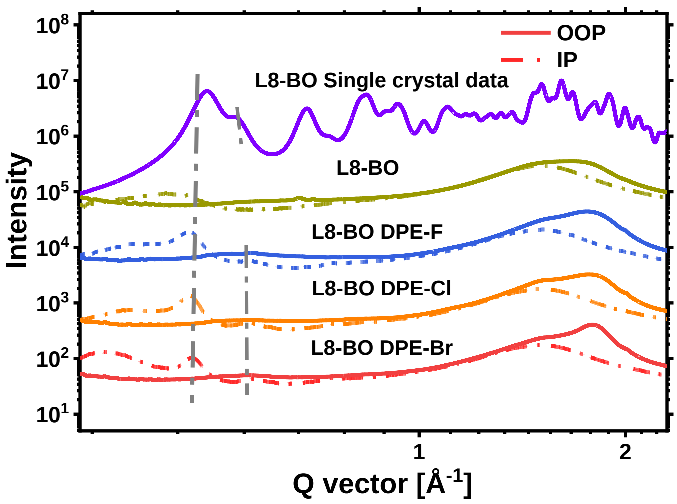

Fig. S7 The curve extracted from single crystal data of L8-BO and the line-cuts extracted from GIWAXS measurements of L8-BO neat films processed without/with DPE-X additives.

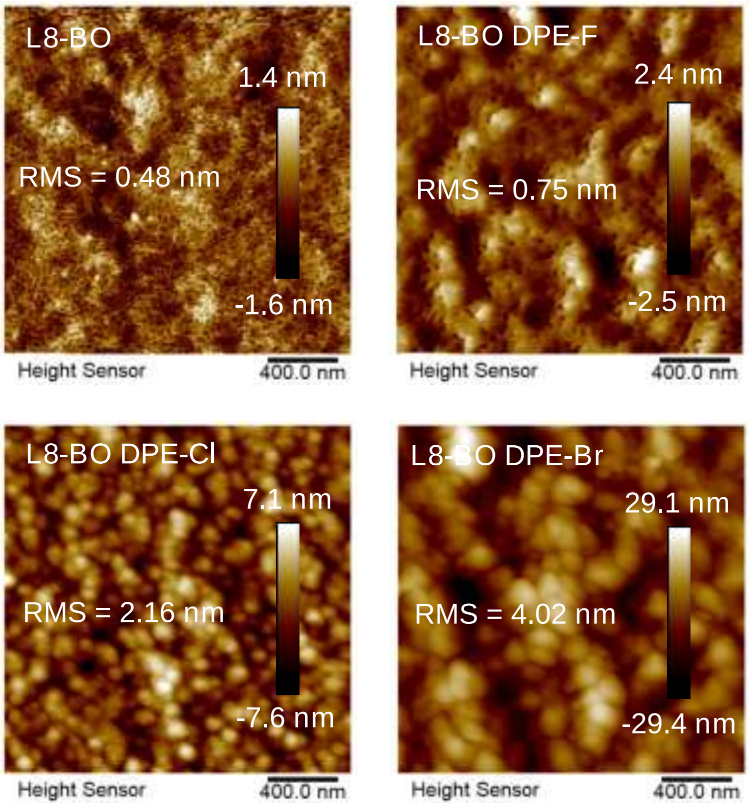

Fig. S8 AFM images of L8-BO films treated without/with DPE-X additives.

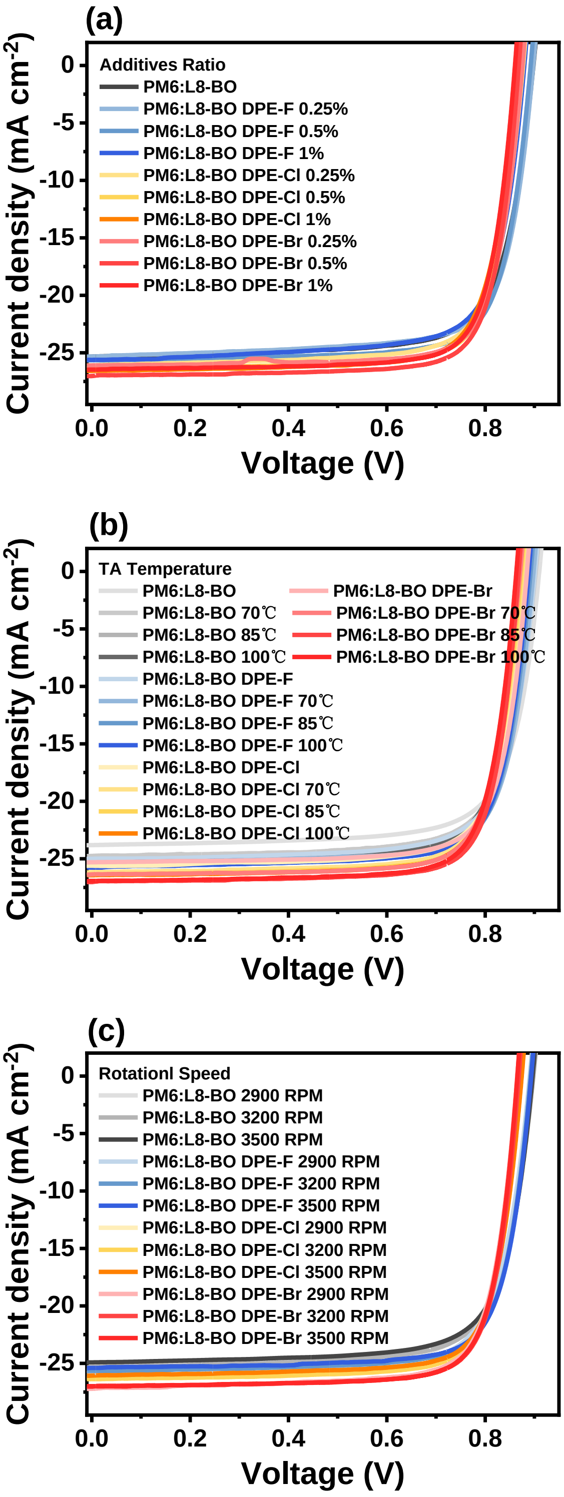

Fig. S9 *J-V* curves of the OSCs based on PM6:L8-BO blends treated without/with DPE-X additives by adjusting the (a) concentration of additives, (b) annealing temperatures, and (c) rotation speed, respectively.

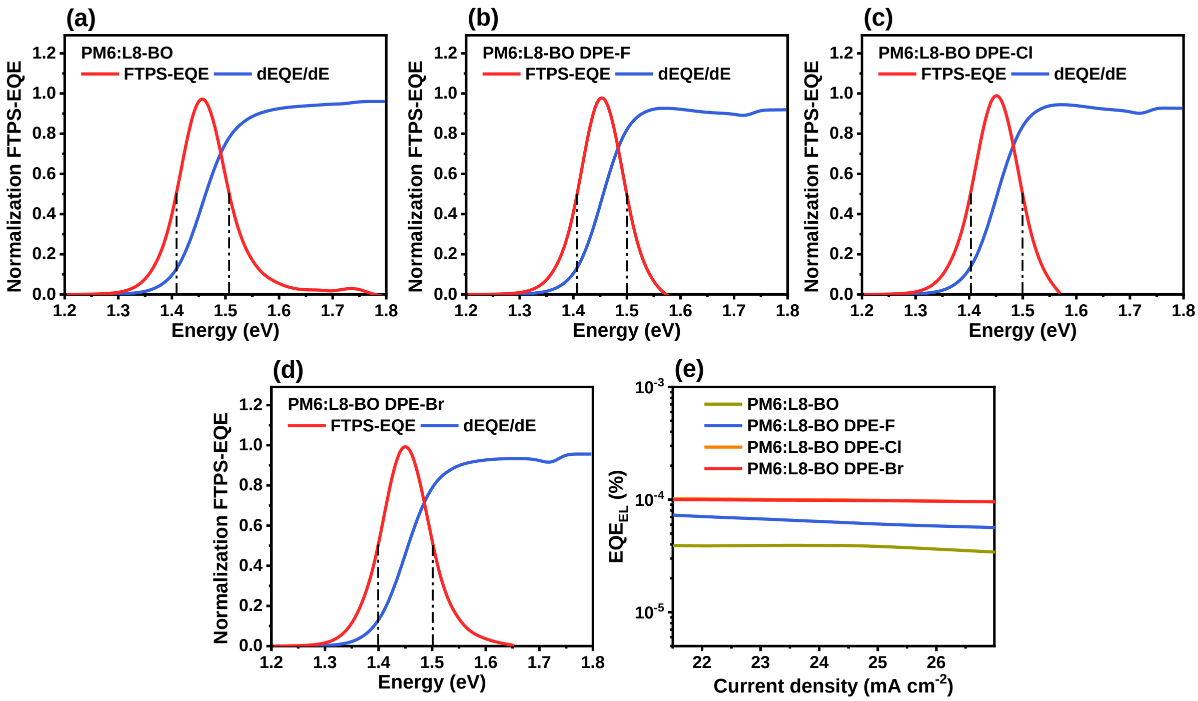

Fig. S10 (a-d) Normalized highly sensitive FTPS-EQE curves and (e) EQE_{EL} curves of the OSCs based on PM6:L8-BO processed without/with DPE-X additives, respectively.

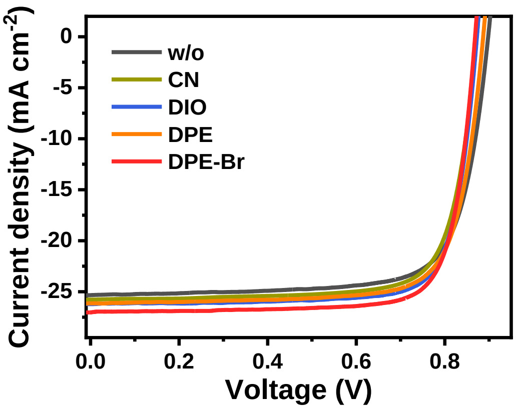

Fig. S11 *J-V* curves of the PM6:L8-BO based OSCs processed based on PM6:L8-BO without/with different liquid additives, respectively.

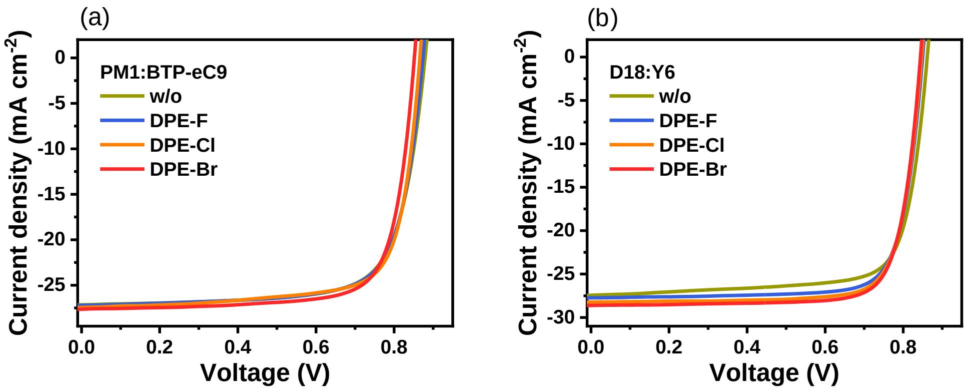

Fig. S12 *J-V* curves of the OSCs based on (a) PM1:BTP-eC9 and (b) D18:Y6 processed without/with DPE-X additives, respectively

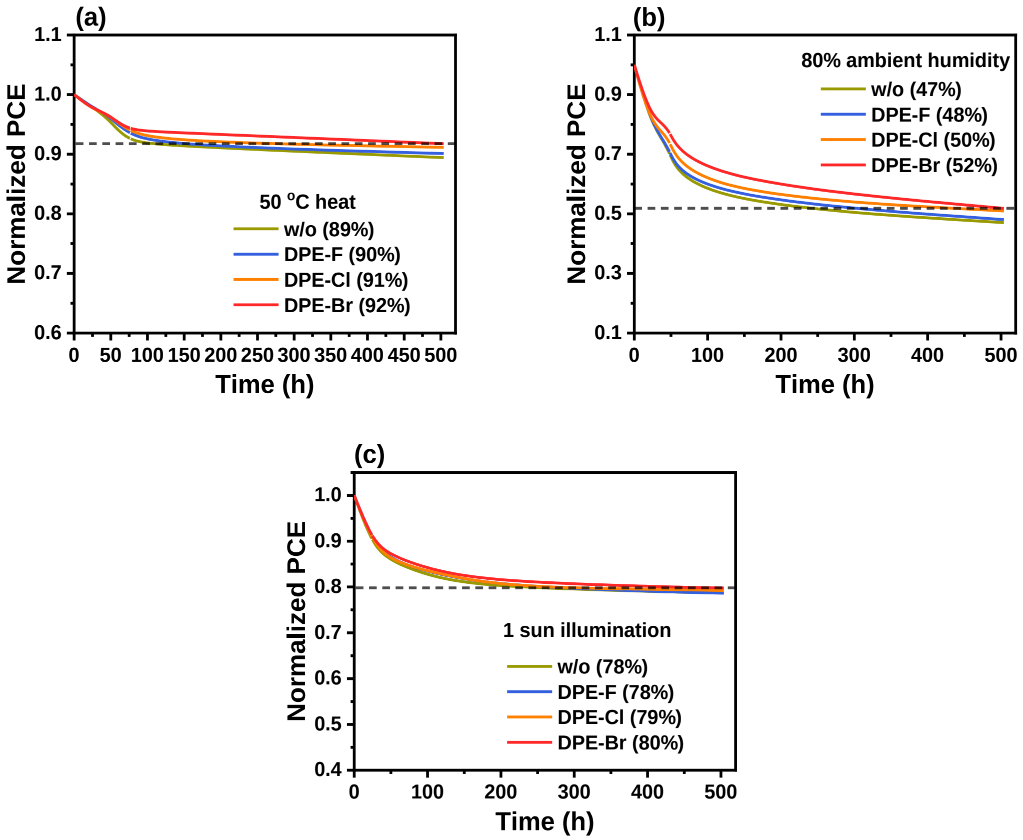

Fig. S13 Normalized PCEs of the OSCs based on PM6:L8-BO processed with additive-free, DPE-F, DPE-Cl, and DPE-Br under (a) thermal annealing at 50 ℃ in N_2-filled glovebox, (b) 80% ambient humidity in air, and (c) illumination simulated at 1 sun intensity in N_2-filled glovebox, respectively.

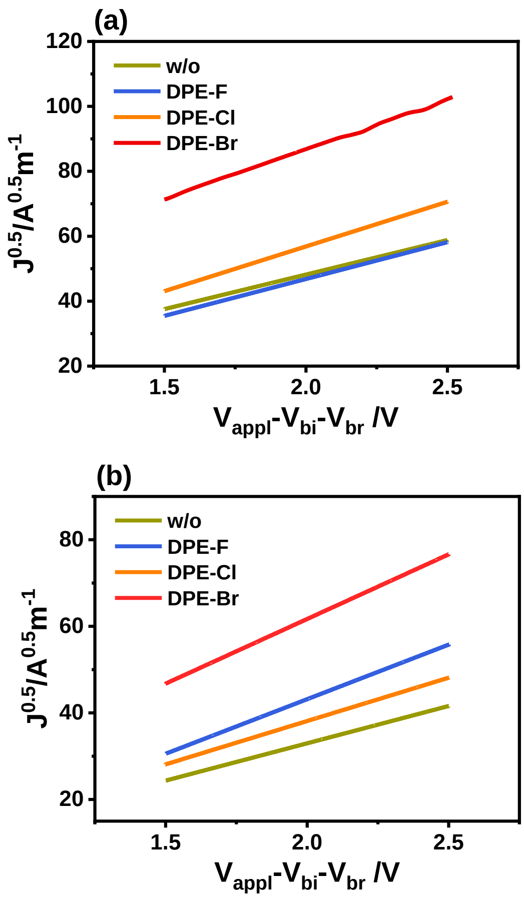

Fig. S14 Current density-applied voltage plots for the (a) hole-only and (b) electron-only devices based on PM6:L8-BO processed without/with DPE-X additives, respectively.

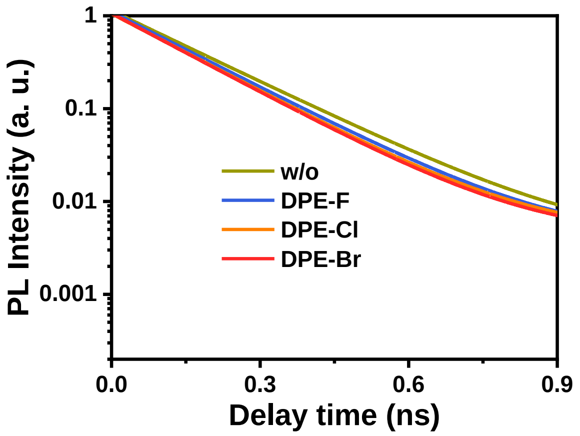

Fig. S15 TRPL spectra of PM6:L8-BO blends processed without/with DPE-X additives.

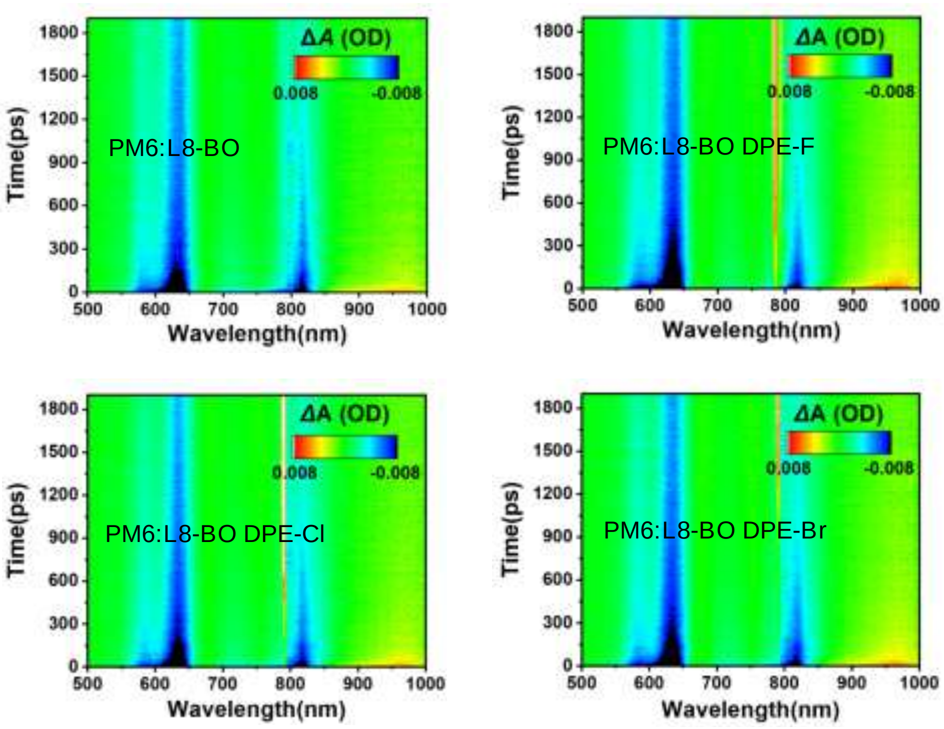

Fig. S16 The fs-TAS spectra of PM6:L8-BO blends treated without/with DPE-X additives under a pump wavelength of 790 nm presented in terms of $\Delta T/T$.

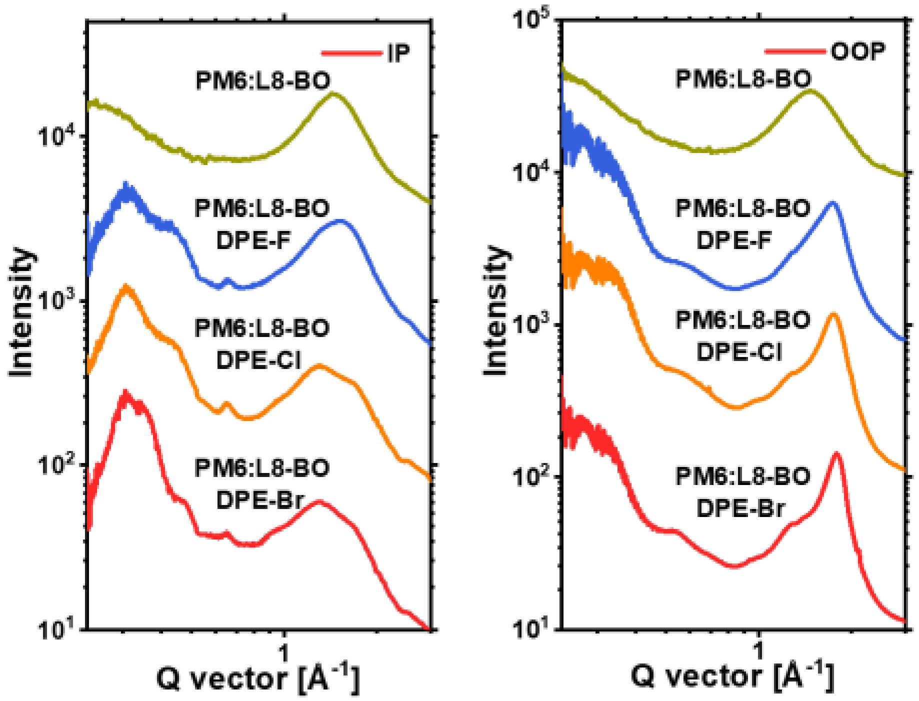

Fig. S17 Line-cut curves from 2D GIWAXS profiles of PM6:L8-BO blends processed without/with DPE-X additives: (a) IP direction and (b) OOP direction.

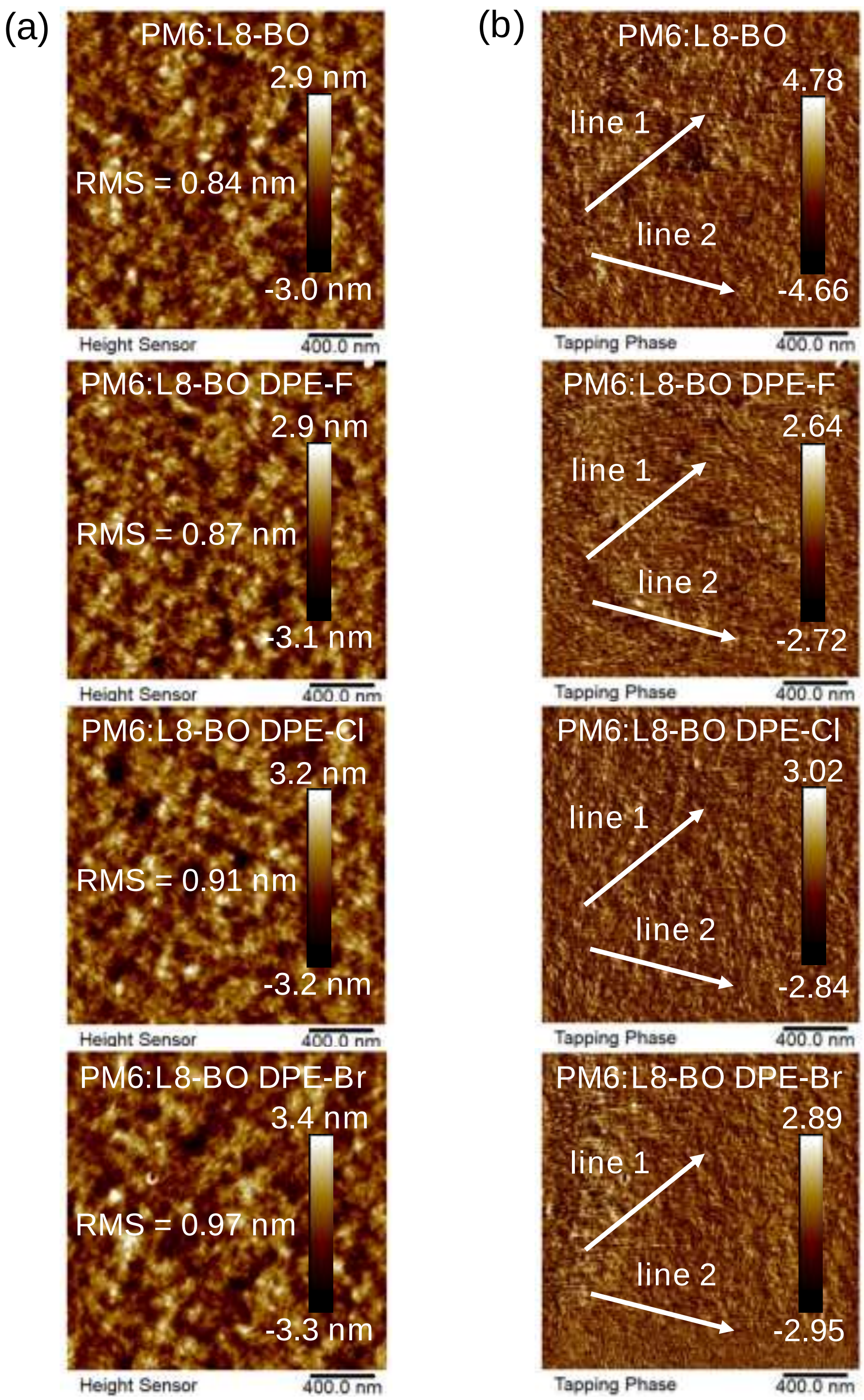

Fig. S18 (a) AFM height images and (b) AFM phase images of the PM6:L8-BO blends processed without/with DPE-X additives.

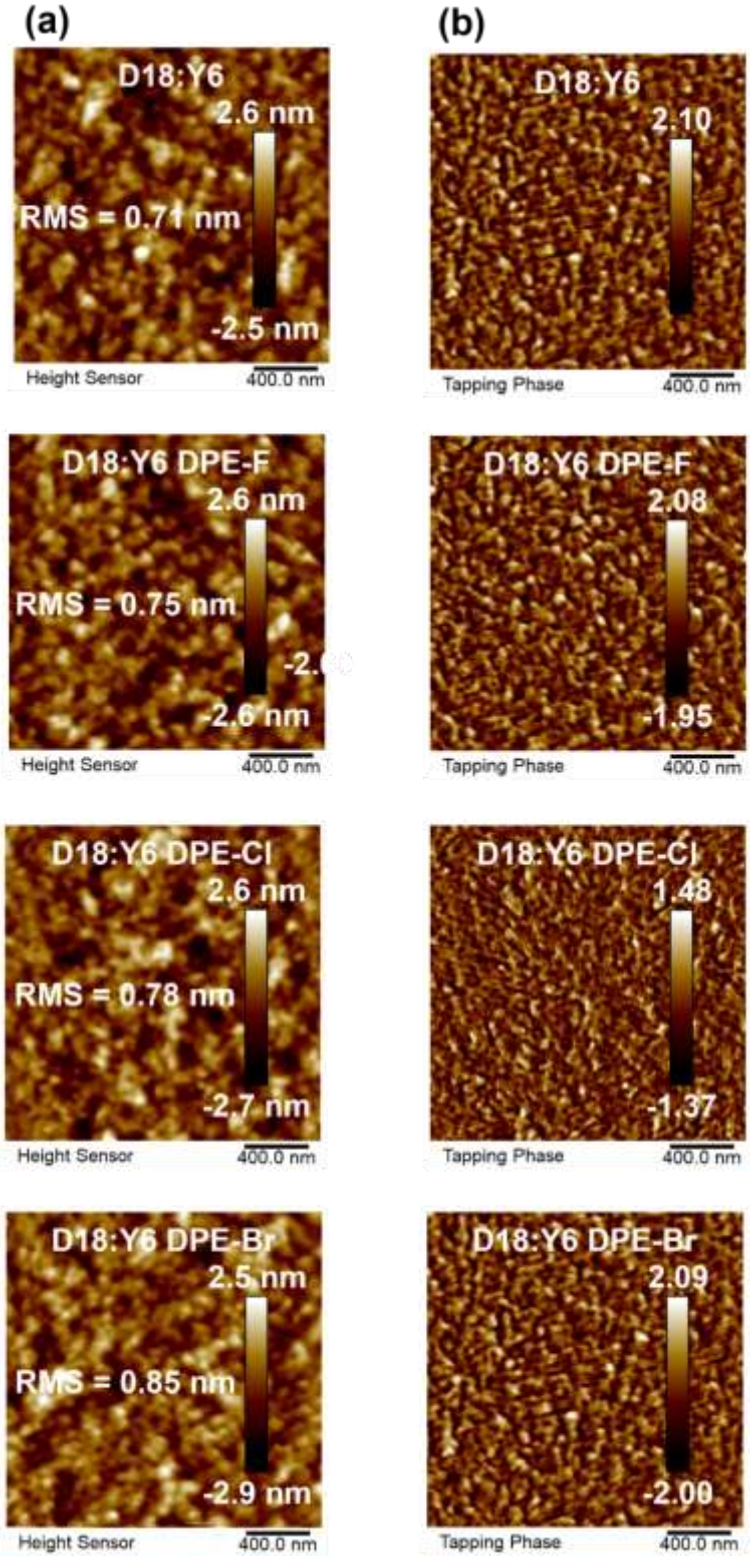

Fig. S19 (a) AFM height images and (b) AFM phase images of the D18:Y6 blends processed without/with DPE-X additives, respectively.

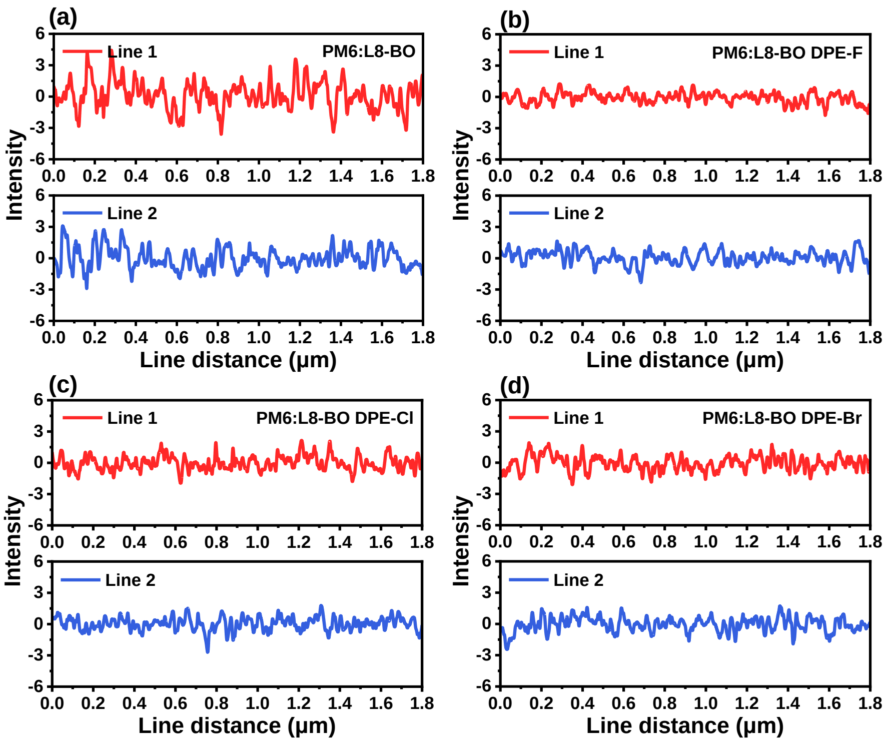

Fig. S20 The line profiles from AFM phase images (labeled the white line) to obtain the FWHM of cross-section for the PM6:L8-BO blends without/with DPE-X additives.

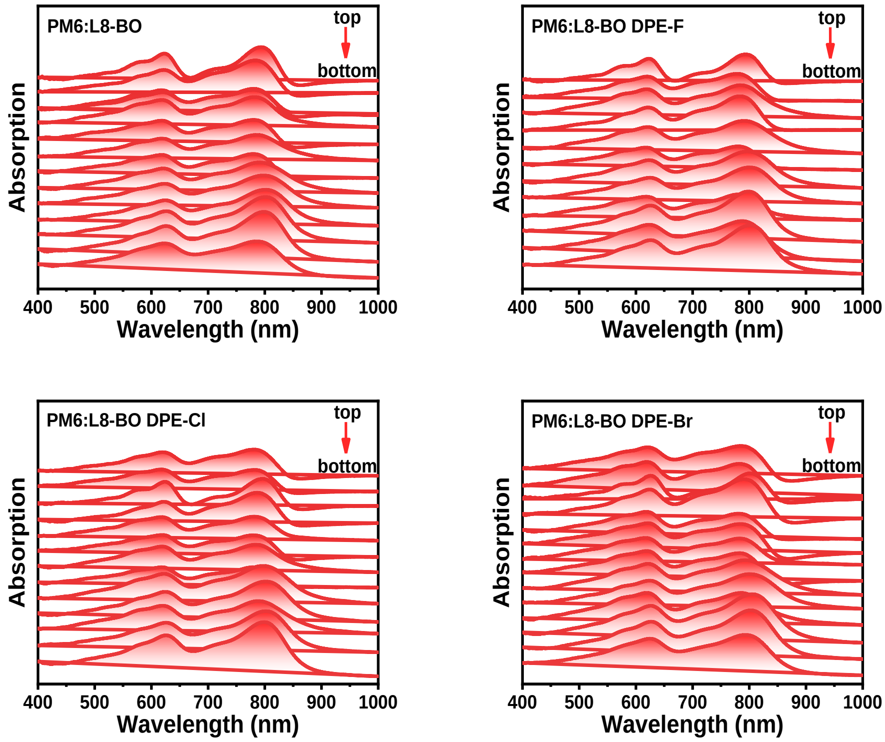

Fig. S21 FLAS images of the PM6:L8-BO blends processed without/with DPE-X additives.

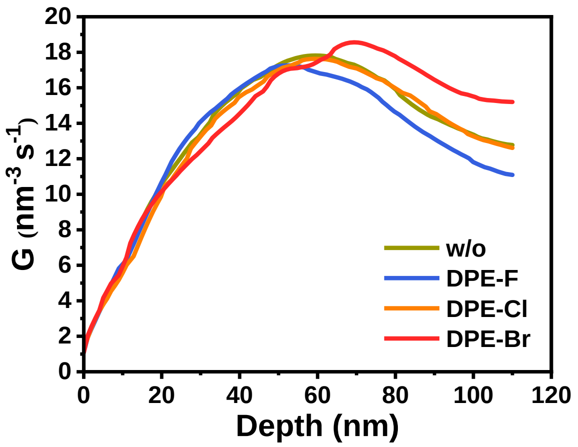

Fig. S22 Dependence of the simulated exciton generation rate (G) on the film depth of blend film for PM6:L8-BO without/with DPE-X additives.

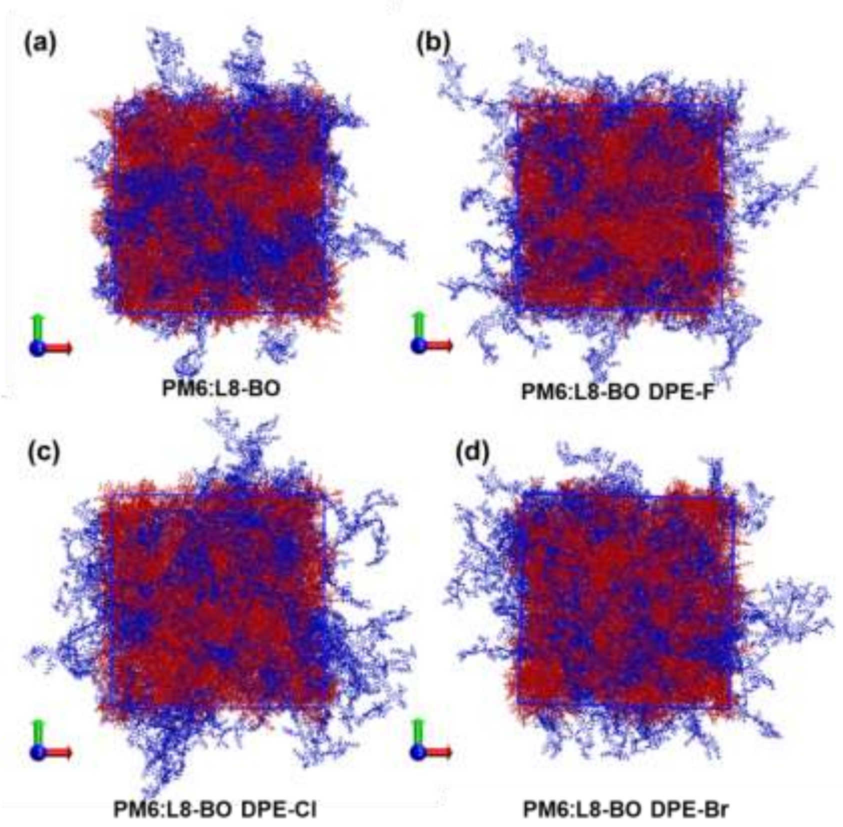

Fig. S23 The final snapshots of the PM6:L8-BO blends processed with (a) additive-free (b) DPE-F, (c) DPE-Cl, and (d) DPE-Br, respectively, wherein the red and blue regions represent the L8-BO and PM6 molecules. RDFs of (d) the L8-BO and BDT unit from PM6, (e) the L8-BO and L8-BO for the simulated blends, respectively.

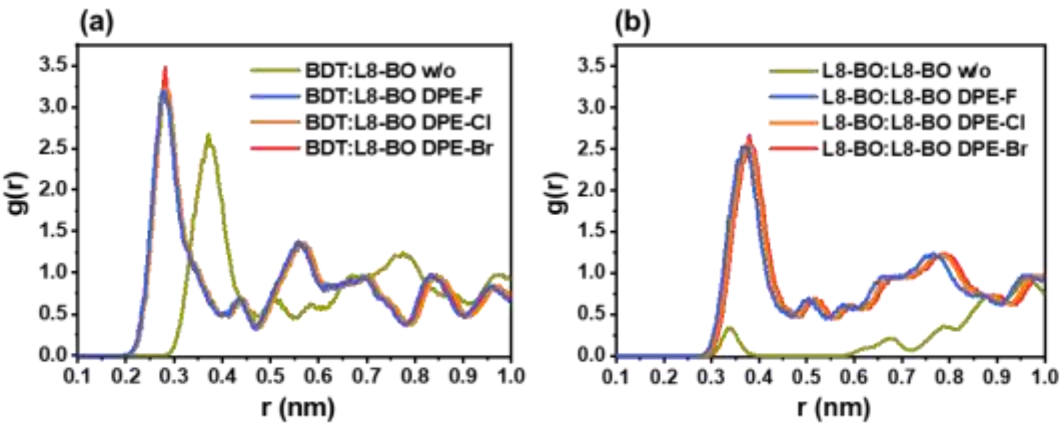

Fig. S24 RDFs of (a) the L8-BO and BDT unit from PM6, (b) the L8-BO and L8-BO for the simulated blends, respectively.

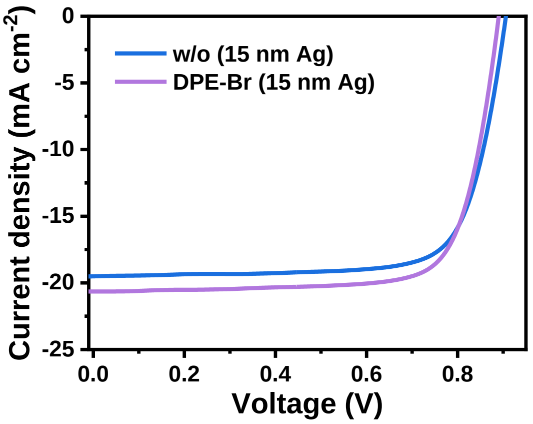

Fig. S25 *J-V* curves of the semitransparent OSCs based on additive-free and DPE-Br treated D18:L8-BO:BTP-eC9 with a device area of 0.038 cm^2.

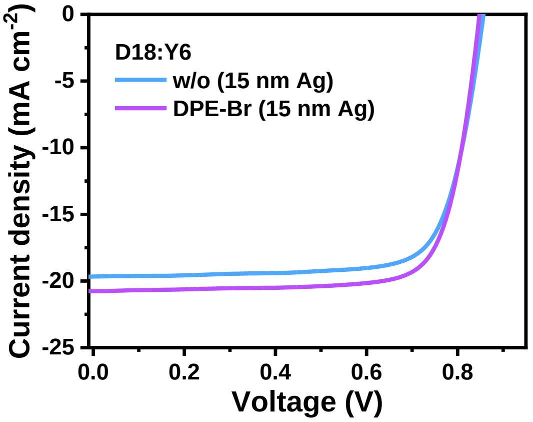

Fig. S26 *J-V* curves of the semitransparent OSCs based on additive-free and DPE-Br treated D18:Y6.

Table S1 Photovoltaic parameters of the state-of-the-art OSCs based on active layers processed different solvents additives.

Active layers	Additives	V_{OC} [V]	J_{SC} [mA cm^{-2}]	FF [%]	PCE [%]	Ref.
PM6:L8-BO	DPE-Br	0.868	26.81	79.08	18.40	**This Work**
D18:L8-BO	DPE-Br	0.902	26.38	79.90	19.01	
**D18:L8-BO: BTP-eC9**	**DPE-Br**	**0.887**	**27.99**	**79.80**	**19.81**	
PM6:L8-BO	DIO	0.869	26.35	79.6	18.23	*Matter,* **2021**, *4*, 2542-2552
PM6:L8-BO	DIM	0.893	26.03	80.0	18.60	
PM6:Y6	FN	0.83	26.98	77.8	17.5	*Energy Environ. Sci.*, **2021**, *14*, 3044-3052
PM6:Y6	CN	0.84	25.79	71.9	15.7	
PM6:Y6	BN	0.84	26.00	74.9	16.4	
PM6:Y6	FBrT	0.85	26.78	78.6	17.7	*Energy Environ. Sci.*, **2022**, *15*, 5137-5148
PM6:L8-BO	ODBB	0.88	25.57	79.20	17.82	*Adv. Energy Mater.*, **2023**, *13*. 2300524
PM6:L8-BO	MDBB	0.89	25.72	79.38	18.17	
PM6:L8-BO	T-2OEH	0.888	26.9	80.4	19.2	*Adv. Mater.*, **2023**, *36*. 2308608
D18:L8-BO	T-2OEH	0.885	26.9	78.6	18.7	
PM6:Y6	DPS	0.825	26.61	76.81	16.86	*Sci. China Chem.*, **2023**, *66*, 1500-1510
PM6:L8-BO	DICO	0.887	26.85	80.1	19.1	*Energy Environ. Sci.*, **2024**, *17*, 1916-1930
PM6:BTP-eC9	DBE	0.86	28	80.5	19.4	*Energy Environ. Sci.*, **2024**, *17*, 5542-5551
PM6:L8-BO	OFIB	0.91	25.66	79.15	18.25	*Adv. Funct. Mater.*, **2024**, *34*, 2310312
PM6:L8-BO: BTP-eC9	Th-ClSi	0.886	27.22	79.5	19.17	*Adv. Funct. Mater.*, **2024**, *34*. 2313744
PM6:L8-BO	DTH	0.861	27.07	79.4	18.51	*Adv. Funct. Mater.*, **2024**, 2401823
PM6:D18-Cl: L8-BO	DTH	0.871	27.44	79.78	19.7	

Table S2 GIWAXS parameters of L8-BO neat films processed with and without additives.

	in-plane					
	location (Å^{-1})	*d*-spacing (Å)	CCL (Å)	location (Å^{-1})	*d*-spacing (Å)	CCL (Å)
L8-BO	0.42	14.9	53.3			
L8-BO DPE-F	0.41	15.4	40.4	0.46	13.6	161.6
L8-BO DPE-Cl	0.40	15.8	44.5	0.46	13.6	176.7
L8-BO DPE-Br	0.35	17.8	85.7	0.47	13.5	226.2
	out-of-plane					
	location (Å^{-1})	*d*-spacing (Å)	CCL (Å)	location (Å^{-1})	*d*-spacing (Å)	CCL (Å)
L8-BO				1.68	3.7	12.2
L8-BO DPE-F	1.49	4.2	15.2	1.78	3.5	16.1
L8-BO DPE-Cl	1.50	4.2	15.5	1.80	3.5	17.5
L8-BO DPE-Br	1.51	4.1	16.0	1.80	3.5	22.6

Table S3 Photovoltaic parameters of the OSCs based on PM6:L8-BO with different treatment processes.

**System**	**DPE-X [%]**	**TA [℃]**	**RPM**	V_{OC} **[V]**	J_{SC} **[mA cm^{-2}]**	**FF [%]**	**PCE [%]**
PM6:L8-BO	w/o	w/o	3200	0.910	23.81	74.78	16.20
	w/o	85	2900	0.897	25.62	73.94	16.99
	**w/o**	**85**	**3200**	**0.898**	**25.34**	**75.06**	**17.08**
	w/o	85	3500	0.897	24.92	75.28	16.73
PM6:L8-BO DPE-F	0.25	85	3200	0.898	25.31	75.93	17.27
	0.5	w/o	3200	0.902	25.00	75.13	16.84
	0.5	70	3200	0.898	25.33	77.44	17.62
	0.5	85	2900	0.890	25.85	76.02	17.51
	**0.5**	**85**	**3200**	**0.892**	**25.68**	**77.41**	**17.73**
	0.5	100	3200	0.888	25.75	77.39	17.71
	0.5	85	3500	0.893	25.37	78.03	17.69

	1	85	3200	0.881	25.69	77.57	17.57
PM6:L8-BO DPE-Cl	0.25	85	3200	0.877	26.08	76.46	17.50
	0.5	w/o	3200	0.884	25.61	76.43	17.20
	0.5	70	3200	0.877	26.16	77.32	17.75
	0.5	85	2900	0.872	26.25	78.47	17.97
	**0.5**	**85**	**3200**	**0.874**	**26.37**	**78.24**	**18.03**
	0.5	100	3200	0.866	26.43	78.27	17.92
	0.5	85	3500	0.874	26.05	78.39	17.86
	1	85	3200	0.867	26.58	77.22	17.79
PM6:L8-BO DPE-Br	0.25	85	3200	0.875	26.11	78.49	17.95
	0.5	w/o	3200	0.878	25.85	77.54	17.48
	0.5	70	3200	0.871	26.37	78.08	17.94
	0.5	85	2900	0.865	27.21	77.73	18.31
	**0.5**	**85**	**3200**	**0.868**	**26.81**	**79.08**	**18.40**
	0.5	85	3500	0.865	26.84	79.08	18.36
	0.5	100	3200	0.863	26.93	78.46	18.25
	1	85	3200	0.860	26.45	78.95	17.97

Table S4 Detailed E_{loss} values of the OSCs based on PM6:L8-BO without/with DPE-X additives.

Additives	E_{g} [eV]	E_{loss} [eV]	qV_{OC}^{SQ} [eV]	qV_{OC}^{rad} [eV]	ΔE_1 [eV]	ΔE_2 [eV]	ΔE_3 [eV][a]	ΔE_3 [eV][b]
w/o	1.457	0.559	1.199	1.155	0.258	0.044	0.257	0.263
DPE-F	1.453	0.561	1.195	1.140	0.258	0.055	0.248	0.252
DPE-Cl	1.450	0.576	1.192	1.112	0.258	0.080	0.238	0.239
DPE-Br	1.449	0.581	1.191	1.107	0.258	0.084	0.239	0.239

[a]ΔE_3 calculated from V_{OC}^{rad}-qV_{OC}. [b]ΔE_3 calculated from the EQE_{EL} measured using a silicon detector.

Table S5 Photovoltaic data of the PM6:L8-BO based OSCs processed without/with different liquid additives.

Active layers	Additives	V_{OC} [V]	J_{SC} [mA cm^{-2}]	FF [%]	PCE[%]
PM6:L8-BO	w/o	0.898	25.34	75.06	17.08
	CN	0.872	25.77	77.01	17.30
	DIO	0.872	26.21	78.76	18.01
	DPE	0.887	26.15	76.46	17.73
	DPE-Br	0.868	26.81	79.08	18.40

Table S6 Photovoltaic data of the OSCs based on PM1:BTP-eC9 and D18:Y6 processed without/with DPE-X additives.

Active layers	Additives	V_{OC} [V]	J_{SC} [mA cm^{-2}]	FF [%]	PCE [%]
PM1:BTP-eC9	w/o	0.879	27.15	73.61	17.47
D18:Y6	w/o	0.862	27.44	76.38	18.17
PM1:BTP-eC9	DPE-F	0.874	27.22	74.23	17.54
D18:Y6	DPE-F	0.850	27.74	78.79	18.67
PM1:BTP-eC9	DPE-Cl	0.866	27.45	75.15	17.73
D18:Y6	DPE-Cl	0.847	28.22	79.06	19.00
PM1:BTP-eC9	DPE-Br	0.852	27.63	76.31	17.91
D18:Y6	DPE-Br	0.845	28.62	79.40	19.29

Table S7 he μ_h and μ_e data of the devices based on PM6:L8-BO processed without/with DPE-X additives.

Active layers	Additives	μ_h [cm^2 V^{-1} s^{-1}]	μ_e [cm^2 V^{-1} s^{-1}]	μ_h/μ_e
PM6:L8-BO	w/o	1.51×10^{-4}	1.09×10^{-4}	1.38
	DPE-F	1.72×10^{-4}	1.47×10^{-4}	1.17
	DPE-Cl	$2.53\times10^{-}$	2.33×10^{-4}	1.09
	DPE-Br	3.42×10^{-4}	3.27×10^{-4}	1.05

Table S8 GIWAXS parameters of PM6:L8-BO binary blends processed with and without additives.

	in-plane					
	location (Å^{-1})	*d*-spacing (Å)	CCL (Å)	location (Å^{-1})	*d*-spacing (Å)	CCL (Å)
PM6:L8-BO	0.23	26.9	23.4	1.46	4.3	10.7
PM6:L8-BO DPE-F	0.31	20.1	43.3	1.49	4.2	8.8
PM6:L8-BO DPE-Cl	0.32	19.6	56.4	1.31	4.8	8.0
PM6:L8-BO DPE-Br	0.32	19.4	57.8	1.30	4.7	6.6
	out-of-plane					
	location (Å^{-1})	*d*-spacing (Å)	CCL (Å)	location (Å^{-1})	*d*-spacing (Å)	CCL (Å)
PM6:L8-BO				1.47	4.3	9.7
PM6:L8-BO DPE-F	0.26	24.0	37.7	1.72	3.6	15.0
PM6:L8-BO DPE-Cl	0.27	23.7	33.0	1.74	3.6	20.3
PM6:L8-BO DPE-Br	0.27	23.4	37.7	1.77	3.5	24.1

Table S9 Efficiency evolution of OSC modules.

Publish date	Active layers	Device structure	Area [cm^2]	PCE [%]	Ref.
-	**D18:L8-BO: BTP-eC9**	**Glass/ITO/PEDOT:PSS/AL/PDIN/Ag**	**11.6**	**16.42**	**This work**
2015.11	PTB7-Th: $PC_{70}BM$	Glass/ITO/ZnO/AL/ MoO_3 /Ag	16.6	6.7	*Nat. Commun.*, **2016**, *7*, 10279
2016.1	PBTZT-stat-BDTT-8:PCBM	Glass/FTO/ZnO/AL/ PEDOT:PSS/Ag	35	5.3	*Energy Environ. Sci.*, **2016**, *9*, 89-94
2019.12	PM6:Y6	Glass/ITO/PEDOT:PSS/AL/ PNDIT-F3N-Br/Ag	11.52	11.86	*Joule*, **2019**, *4*, 407-419
2019.8	PBDB-T:ITIC	PET/ITO/ZnO/AL /MoO_3 /Ag	15	8.9	*Adv. Mater.*, **2019**, *31*, 1903649
2020.7	PBDB-T:ITIC	Glass/ITO/ZnO/AL /MoO_3 /Ag	58.5	9.03	*Nano Energy*, **2020**, *77*, 105147
2020.8	PM6:DTY6	Glass/ITO/PEDOT:PSS/AL/ PNDIT-F3NBr/Ag	18	14.4	*Joule*, **2020**, *4*, 2004-2016
2020.11	PTB7-Th:COi8DFIC:PC71BM	PET/Ag-grids/PH1000/ ZnO/AL/MoO_3 /Ag	50	9.05	*Adv. Mater.*, **2020**, *32*, 2005153
2021.11	TPD-3:Y6	Glass/ITO/ZnO/AL/ MoO_3 /Ag	4.08	9.31	*Adv. Energy Mater.*, **2021**, *11*, 2102648
2021.12	PM6:BTP-eC9	Glass/ITO/PEI-Zn/AL/MoO_3 /Ag	25.21	14.07	*Adv. Funct. Mater.*, **2022**, *32*, 2110209
2021.8	PBDB-T-2F:Y6:PC71BM	PET/Ag-grid/AgNWs/ PEI-Zn/AL/MoO_3 /Ag	10.1	12.6	*Adv. Mater.*, **2021**, *33*, 2103017
2022.4	PM6:Y6:PC71BM	Glass/AgNWs/PEI-Zn /AL/PEDOT:F/AgNWs	12.2	13.07	*Nat. energy*, **2022**, *7*, 352-359
2022.6	PM6:BTP-Bo-4Cl	Glass/ITO/PEDOT:PSS/AL/ Bis-FIMG/Ag	19.3	14.35	*Adv. Mater.*, **2022**, *34*, 2110569
2023.8	PM6:CH8-4	Glass/ITO/ ZnO/AL/ MoO_3/Ag	2.88	13.59	*Angew. Chem. Int. Ed.*, **2023**, *62*, e202307962
2024.3	PM6:D18:L8-BO	Glass/ITO/AZO/AL/ MoO_3/Al	15.64	16.03	*Energy Environ. Sci.*, **2024**, *17*, 2935-2944
2024.3	PM6:L8-BO:PC61BM	Glass/ITO/ZnO/AL/ MoO_3/Ag	11.08	16.1	*Cell Rep. Phys. Sci.*, **2024**, *5*, 101883
2024.4	PM6:L8-BO: BTO-BO	Glass/ITO/PEDOT:PSS/AL/ C60/BCP/Ag	15.03	16.35	*Adv. Mater.*, **2024**, *36*, 2402350
2024.5	PM6:D18: BTP-eC9	Glass/ITO/PEDOT:PSS /C60/BCP/Ag	15.64	16.7	*Giant*, **2024**, *18*, 100286
2024.7	PM6:N-HD	Glass/ITO/ZnO NPs/ AL/MoO_3/Ag	55	12.2	*Adv. Sci.*, **2024**, *11*, 2405716
2024.8	D18-Cl:BTP-4F-P2EH	Glass/ITO/PEDOT:PSS/AL/ PDINN/Ag	17.6	17	*Joule*, **2024**, *8*, 3153-3168
2024.9	PTzBI-Cl:DT-Y6:BTR-Cl	Glass/ITO/PEDOT:PSS/AL/ PNDIT-F3N/Ag	100	11	*Energy Environ. Sci*. **2024**, *17*, 7681-7690

Table S10 Photovoltaic data of the ST-OSCs based on D18:Y6 processed without/with DPE-Br.

Additive	V_{OC} [V]	J_{SC} [mA/cm^2]	FF [%]	PCE [%]
w/o	0.857	19.66	75.92	12.82
DPE-Br	0.848	20.76	77.19	13.62

References

[S1]Li C, Zhou J, Song J et al. Non-fullerene acceptors with branched side chains and improve molecular packing to exceed 18% efficiency in organic solar cells. *Nat Energy* 2021; **6**: 605-13.

[S2]Cui Y, Yao H, Zhang J et al. Single-Junction Organic Photovoltaic Cells with Approaching 18% Efficiency. *Adv Mater* 2020; **32**: 1908205.

[S3] Yang C, Liu D, Bates M et al. Planning a Low-Carbon Energy Transition: What Can and Can't the Models Tell Us? *Joule* **2019**; 3: 1795-8.
